# CD44/CD44v6 a Reliable Companion in Cancer-Initiating Cell Maintenance and Tumor Progression

**DOI:** 10.3389/fcell.2018.00097

**Published:** 2018-08-28

**Authors:** Zhe Wang, Kun Zhao, Thilo Hackert, Margot Zöller

**Affiliations:** ^1^Department of Oncology, First Affiliated Hospital of Guangdong Pharmaceutical University, Guangdong, China; ^2^Pancreas Section, University Hospital of Surgery, Heidelberg, Germany

**Keywords:** cancer initiating cells, CD44, apoptosis resistance, EMT, migration, metastasis, tumor exosomes

## Abstract

Metastasis is the leading cause of cancer death, tumor progression proceeding through emigration from the primary tumor, gaining access to the circulation, leaving the circulation, settling in distant organs and growing in the foreign environment. The capacity of a tumor to metastasize relies on a small subpopulation of cells in the primary tumor, so called cancer-initiating cells (CIC). CIC are characterized by sets of markers, mostly membrane anchored adhesion molecules, CD44v6 being the most frequently recovered marker. Knockdown and knockout models accompanied by loss of tumor progression despite unaltered primary tumor growth unraveled that these markers are indispensable for CIC. The unexpected contribution of marker molecules to CIC-related activities prompted research on underlying molecular mechanisms. This review outlines the contribution of CD44, particularly CD44v6 to CIC activities. A first focus is given to the impact of CD44/CD44v6 to inherent CIC features, including the crosstalk with the niche, apoptosis-resistance, and epithelial mesenchymal transition. Following the steps of the metastatic cascade, we report on supporting activities of CD44/CD44v6 in migration and invasion. These CD44/CD44v6 activities rely on the association with membrane-integrated and cytosolic signaling molecules and proteases and transcriptional regulation. They are not restricted to, but most pronounced in CIC and are tightly regulated by feedback loops. Finally, we discuss on the engagement of CD44/CD44v6 in exosome biogenesis, loading and delivery. exosomes being the main acteurs in the long-distance crosstalk of CIC with the host. In brief, by supporting the communication with the niche and promoting apoptosis resistance CD44/CD44v6 plays an important role in CIC maintenance. The multifaceted interplay between CD44/CD44v6, signal transducing molecules and proteases facilitates the metastasizing tumor cell journey through the body. By its engagement in exosome biogenesis CD44/CD44v6 contributes to disseminated tumor cell settlement and growth in distant organs. Thus, CD44/CD44v6 likely is the most central CIC biomarker.

## Introduction

CD44/CD44 variant isoforms (CD44v) are adhesion molecules also described as most prominent function-relevant cancer initiating cell (CIC) markers (Zöller, 2011; Yan et al., 2015). To shed light on the engagement of CD44/CD44v6 in CIC activities, we will first introduce the CD44 molecule, CIC and exosomes (Exo) and then outline the state of knowledge on the linkage between CD44/CD44v6 and CIC with emphasis on the requirement of a niche (Prasetyanti et al., 2013), apoptosis resistance (Ramdass et al., 2013; Colak and Medema, 2014; Vlashi and Pajonk, 2015), epithelial mesenchymal transition (EMT) (Dontu and Wicha, 2005; Wells et al., 2011) and tumor progression (Elshamy and Duhé, 2013). Finally, the contribution of CD44/CD44v6 to metastatic settlement being promoted by tumor exosomes (TEX), which are suggested to transfer CIC-features to Non-CIC, to promote angiogenesis, to prepare a premetastatic niche and to modulate hematopoiesis toward an immunosuppressive phenotype (Hannafon and Ding, 2015; Minciacchi et al., 2015), will be discussed.

### CD44

#### The CD44 molecule

**Figure 1:**
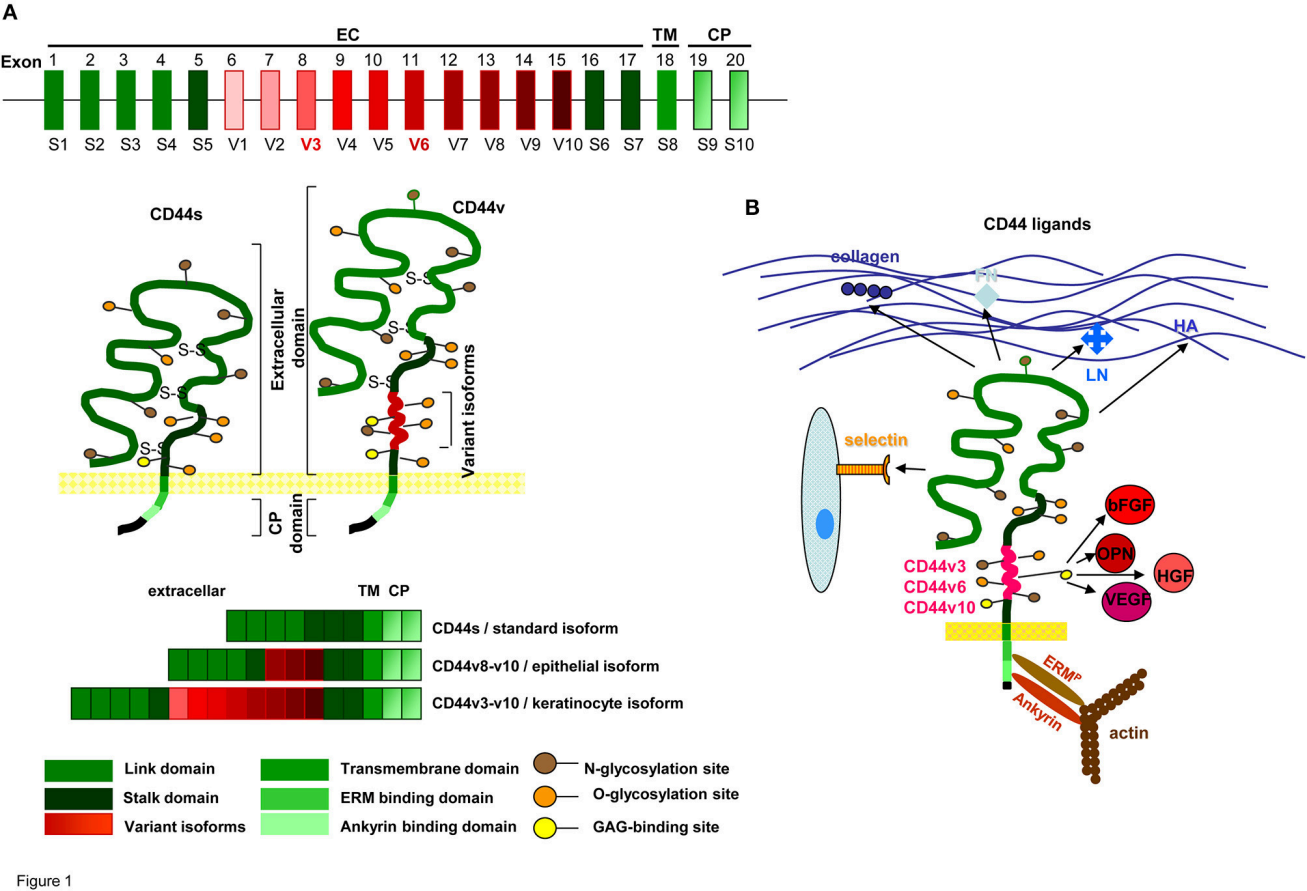
CD44 molecules, prominent ligands and associated molecules.**(A)** Genomic organization and protein structure of CD44s and CD44v, glycosylation sites, the location in the cell membrane and some frequently observed CD44v exon product combinations are shown. **(B)** Most prominent matrix protein and cellular ligands of the globular N-terminal domains and the binding sites of the C-terminal domain to the cytoskeletal linker proteins ERM and ankyrin are indicated. Only CD44v3, CD44v6, and CD44v10 have binding sites for cytokines and chemokines.

CD44 is a type I transmembrane glycoprotein that varies in size due to *N*- and *O*-glycosylation and insertion of alternatively spliced exon products (Idzerda et al., 1989; Goldstein and Butcher, 1990; Screaton et al., 1992). The hematopoietic isoform (CD44s) has seven extracellular domains, a transmembrane, and a cytoplasmic domain encoded by exons 9 or 10 (Peach et al., 1993). Up to 10 variant exon products can be inserted by alternative splicing between exons 5 and 6 (Screaton et al., 1992). CD44 is a member of the cartilage link protein family (Idzerda et al., 1989). The globular structure of the *N*-terminal region is stabilized by conserved cysteins. Two cysteins in the flanking region account for link domain folding (Ishii et al., 1993). The globular domain are followed by exon products 5–7, which are heavily glycosylated, form a stalk like structure and contain putative proteolytic cleavage sites (Neame and Isacke, 1993; Ruiz et al., 1995). Variable exon products are inserted in this region (Bennett et al., 1995). Whereas CD44s is expressed by most cells, CD44v is expressed only on subpopulations of epithelial and hematopoietic cells, particularly during embryogenesis and hematopoiesis, on leukocytes during activation and frequently on CIC (Ruiz et al., 1995). Insertion of CD44v exon products is variable, but some combinations, i.e., the keratinocyte isoform (v8-v10) and the epidermal isoform (exons v3-v10) are preferentially recovered in selective tissues (Ruiz et al., 1995). The transmembrane region supports CD44 oligomerization and recruitment into glycolipid-enriched membrane domains (GEM). The GEM location is utmost important for the interaction of CD44 with extracellular ligands and the association with other transmembrane and cytoplasmic molecules (Liu and Sy, 1997; Föger et al., 2001). The cytoplasmic tail contains binding sites for cytoskeletal proteins (Lokeshwar et al., 1994; Oliferenko et al., 1999) (Figure 1A).

Briefly, CD44s is a heavily glycosylated transmembrane protein expressed by most epithelial and mesenchymal cells. CD44v is expressed in selected tissues, but is frequently upregulated in CIC.

#### CD44 ligands

The CD44 link domain contains binding sites for collagen, laminin (LN), fibronectin (FN), E- and L-selectin (Jalkanen and Jalkanen, 1992; Toyama-Sorimachi and Miyasaka, 1994; Konstantopoulos and Thomas, 2009). CD44 is the major hyaluronan (HA) receptor. HA binds to a basic motif outside the link domain (Aruffo et al., 1990). Not all CD44+ cells bind HA, but binding being induced by CD44 cross-linking, indicates conformation-dependent HA-binding (Suzuki et al., 2015). CD44 has two additional binding sites for glycosaminoglycans (GAG) (Greenfield et al., 1999; Higman et al., 2014). The cytoplasmic tail binds the cytoskeletal linker proteins ankyrin and ezrin, radixin, moesin (ERM) (Lokeshwar et al., 1994; Mori et al., 2008). Ankyrin contacts spectrin and is involved in HA-dependent adhesion and motility (Lokeshwar et al., 1994). ERM proteins regulate migration, cell shape and protein resorting (Fehon et al., 2010; Stamenkovic and Yu, 2010). Activated ERM proteins bind with the *N*-terminus a motif between the transmembrane region and the ankyrin binding site of CD44: The C-terminus binds to F-actin (Mori et al., 2008). The ERM family protein Merlin binds CD44, but lacks the actin-binding domain (Stamenkovic and Yu, 2010). By the binding to cytoskeletal linker proteins the range of CD44-mediated functions expands toward downstream signaling pathways (Mori et al., 2008; Fehon et al., 2010; Bourguignon et al., 2014).

Several of the CD44v isoforms contain post-translational modifications. CD44v3 has a heparan-sulfate (HS) site, promoting growth factor binding (Bennett et al., 1995); CD44v6 binds the hepatocyte growth factor (HGF), the vascular endothelial growth factor (VEGF), and osteopontin (OPN) (Kim et al., 2005; Orian-Rousseau and Ponta, 2008; Tremmel et al., 2009; Yuan et al., 2013). The latter also binds to CD44v10 (Erb et al., 2014). Via cytokine/chemokine binding, CD44v becomes engaged in receptor tyrosine kinase (RTK) activation (Orian-Rousseau, 2015) (Figure 1B).

Thus, CD44 has beside HA additional cellular and extracellular matrix (ECM) ligands. The cytoplasmic tail promotes a linkage to the cytoskeleton and CD44v gains access to transmembrane receptors by cytokine and chemokine binding.

#### CD44 and CD44v6 associated molecules

Of central importance to understand the multitude of CD44/CD44v activities are the associations with several RTK, proteases, and ATP-binding cassette (ABC) transporters.

Through HGF binding, CD44v6 comes into proximity of MET and promotes MET activation, which requires the interaction of the CD44 cytoplasmic tail with ERM proteins for Ras-MAPK pathway activation (Orian-Rousseau et al., 2007). The interaction between CD44v6, MET and ERM proteins is vital, as MET-haploinsufficient CD44 knockout (ko) mice die at birth by defects in synaptogenesis and axon myelination (Matzke et al., 2007). CD44v6-ECM binding also promotes PI3K/Akt pathway activation (Krause and Van Etten, 2005; Weber, 2008) and MET transcription (Adamia et al., 2005). CD44v6 crosslinking similarly affects insulin-like growth factor 1 receptor (IGFR1) and platelet-derived growth factor receptor (PDGFR) activation (Misra et al., 2006). The CD44-RTK interaction can also proceed through proteases. CD44-recruited MMP7 cleaves the proform of the heparin-binding EGF-like growth factor (HBEGF), which binds to CD44v3 (Bennett et al., 1995). Cleaved HBEGF binds ERBB4, which initiates anti-apoptotic signaling cascades (Lynch et al., 2007).

CD44 also associates with G-protein-coupled receptors (GPCR), e.g., CXCR4 (Katoh, 2017), with ABC transporters (Grass et al., 2014; Kozovska et al., 2014) and additional antiapoptotic proteins (Bates et al., 1998; Ghatak et al., 2005; Bourguignon, 2008), with membrane bound proteases such as MMP14 and hyaluronidase (Hyal)2 (Heldin et al., 2014). Finally, CD44 associates with EMT-related transcription factors, e.g., the Wnt signaling pathway via CD44-associated LRP6 (LDL receptor related protein6) (Xu et al., 2015). Of central importance for the engagement of CD44 as a CIC marker are the associations with non-RTK (Cooper and Qian, 2008; Nastase et al., 2017), which can proceed via activated RTK, GPCR, cytoskeletal linker proteins, or directly via GEM-recruited CD44v (Ingley, 2008; Ekyalongo et al., 2015; Korcsmaros et al., 2017).

The impact of CD44 ligand binding and lateral associations on CIC activities are discussed in detail below for relevant contributions to CIC maintenance, apoptosis resistance, EMT, and the metastatic cascade.

#### CIC characterization

Cancer research received a significant hub by the identification of CIC, also defined as cancer stem cells. CIC are a minor subpopulation within the primary tumor mass, yet are suggested to account for tumor initiation, propagation, recurrence, metastasis, and drug- and radiation resistance (Elshamy and Duhé, 2013; Kozovska et al., 2014).

Stem cells (SC) have the capacity to self renew and to differentiate and are drug and radiation resistant. SC are located in special niches, where they receive information from the surrounding matrix, which are essential in stem cell maintenance (Morrison and Scadden, 2014; Rojas-Ríos and González-Reyes, 2014; Tan and Barker, 2014; Vaidya and Kale, 2015; Katoh, 2017). This also accounts for adult SC, well-elaborated for hematopoietic SC (HSPC) in the osteogenic and the vascular niche (Vaidya and Kale, 2015). Virchow's idea that cancer may arise from embryonic-like cells (Virchow, 1858), was first verified for human leukemia-initiating cells (LIC) developing tumors in immunocompromised mice (Lapidot et al., 1994). Leukemia recurrence was prevented by deletion of the HSC factor Bmi1 (Lessard and Sauvageau, 2003). The idea of LIC/CIC was fostered by the recovery of Oct4, Sox2, and Nanog, master transcription factors of embryonic SC (Tárnok et al., 2010), by changes in chromatin organization and epigenetic signatures, important for self renewal and differentiation (Kashyap et al., 2009; Suzuki et al., 2009; Gupta et al., 2010; Messaoudi-Aubert et al., 2010) and by the upregulation of Notch, Wnt, and Hedgehog (HH), which contribute to cell fate decision (Cerdan and Bhatia, 2010).

CIC share with SC apoptosis resistance and altered expression of transcription factors that contribute to EMT and possibly, though still controversially discussed, the requirement of a niche. There is evidence for CD44/CD44v6 supporting CIC in fulfilling these tasks.

#### CIC and tumor progression

Cancer-related death is mostly due to metastasis formation (Sun and Ma, 2015), where tumor cells leave the primary tumor mass, invade the circulation, adhere to the vessel wall, settling and growing in a distinct organ (Seyfried and Huysentruyt, 2013; Samatov et al., 2015). In advance of settling in distant organs, most tumors set metastasis in the draining lymph node, which relies on favored invasion into lymphatic vs. blood vessels. The lymphatic route of metastasizing tumor cells accounts particularly for gastrointestinal cancer and is not solely a sequel of easier access due to a missing or incomplete basement membrane. Searching for differentially expressed genes in metastasizing tumor cells revealed that proteins engaged in (lymph)angiogenesis were dominating and that distinct protein pattern were recovered in tumor cells metastasizing vs. blood or lymphatic vessels (Karaman and Detmar, 2014; Li and Li, 2015). Nonetheless, invasion into lymphatic or blood vessels requires de-anchoring of individual tumor cells from the surrounding cells, one of the processes linked to CIC (Ombrato and Malanchi, 2014; Qiao et al., 2015; Iftakhar-E-Khuda et al., 2016). Finally, settling in distant organs is a non-random process, most cancer being characterized by metastatic site preference (Obenauf and Massagué, 2015). This is facilitated by metastasizing tumor cells creating a niche in the metastatic organ in advance of arrival, the so called “premetastatic niche” (Fessler et al., 2013; Chang et al., 2015). In the absence of tumor cells, the niche can only by formed by tumor-derived factors, which task is mostly undertaken by CIC-derived TEX (Thuma and Zöller, 2014; Giovannetti et al., 2017).

In brief, besides contributing via EMT to the release of tumor cells from the primary tumor mass, CIC are engaged in metastatic settlement, which is supported by CD44/CD44v6. In addition, there is evidence for CD44v6 modulating CIC-TEX during biogenesis, Exo/Exo biogenesis being briefly introduced.

### Exosomes

Exo, small 40–100 nm vesicles, are delivered by many cells and abundantly by CIC (Vlassov et al., 2012) and distribute in the body (Boukouris and Mathivanan, 2015). Exo components are function competent and delivery of their messages (Valadi et al., 2007; Lo Cicero et al., 2015) can modulate targets and reprogram target cells (Hao et al., 2006; Grange et al., 2011; Abd Elmageed et al., 2014; Meseure et al., 2014; Lo Cicero et al., 2015).

Exo biogenesis starts with the formation of early endosomes (EE), deriving from the trans-Golgi network or from internalized membrane microdomains, such as clathrin-coated pits, GEM, and cholesterol- and ceramide-rich compartments (Colombo et al., 2014). The different types of EE are guided toward multivesicular bodies (MVB) by distinct transport machineries (van Niel et al., 2011). During inward budding of so called intraluminal vesicles (ILV) into MVB, vesicles receive their cargo (Colombo et al., 2014; Villarroya-Beltri et al., 2014; Choi et al., 2015; Abels and Breakefield, 2016). MVB are guided toward and fuse with the plasma membrane. The released vesicles are called Exo (Colombo et al., 2014; Abels and Breakefield, 2016).

During invagination into MVB, the small plasma of ILV receives its cargo. Loading is a selective process. Sphingosine-1-phosphatase and diaglycerol are engaged in cargo sorting. Lysophosphatidic acid together with Alix and heat shock protein (HSP)70 promotes inward budding of ILV (Nabhan et al., 2012). Mono-ubiquitination, acylation or myristoylation (Kajimoto et al., 2013), or higher order oligomerization (Rana et al., 2011; Shen et al., 2011) or sphingolipids forming ceramide (Guo et al., 2015) facilitate protein sorting. Annexin-II supports RNA sorting (Vedeler et al., 2012). By coupling of RISC (RNA-induced silencing complex) and a specific EXOmotif binding to hnRNPA2B1 (heterogeneous ribonucleoprotein A2B1), miRNA becomes recruited (Villarroya-Beltri et al., 2013). Long non-coding (lnc)RNA are also selectively captured (Villarroya-Beltri et al., 2013).

Exosomes are composed of a lipid bilayer containing transmembrane proteins. The vesicle lumen encloses proteins, mRNA, non-coding RNA, and DNA.

The lipid envelop is enriched in sphingomyelin, cholesterol, GM3, and phosphatidylserine (Subra et al., 2010). The most abundant constitutive Exo proteins are tetraspanins (Zöller, 2009; Mathivanan et al., 2010; Choi et al., 2012; Rana et al., 2012; Yue et al., 2015). Additional constitutively expressed proteins are structural components and proteins involved in biogenesis including trafficking. Cell type-specific Exo proteins are most comprehensively explored for CIC. All described CIC markers such as MART1, EGFRVIII, MDR1 (multidrug resistance gene 1), EpCAM, MET, mutant KRAS, tissue factor, and CD44/CD44v6 are recovered in TEX (Al-Nedawi et al., 2008; Corcoran et al., 2012; Park et al., 2012; Demory Beckler et al., 2013; Ji et al., 2013; Kumar et al., 2015; Zöller, 2016). The recovery of CIC markers in TEX extends their suggested contribution to tumor progression.

In concern about DNA, RNA and non-coding RNA, most information is available on miRNA. Uncovering that miRNA is linked to prognosis, progression, local recurrence (Sato-Kuwabara et al., 2015), and contributes to EMT (Garg, 2015), fostered progress in oncology. In concern about CD44, miR-34a overexpression inhibits metastasis by regulating CD44 and miR-340 suppresses invasion and metastasis by regulating CD44-associated c-Met and metalloproteinases (MMP) 2 and 9 (Liu et al., 2011; Wu et al., 2011). LncRNA also affects tumor growth and progression. Thus, MALAT-1 promotes tumor growth and migration and prevents tumor cell apoptosis, linc-POU3F3 induces angiogenesis, ZFAS1 promotes proliferation and migration of cancer cells. Interestingly, co-existence of U1 and U2 ribonucleoproteins and cognate snRNA is supposed indicating a link to splicing events in recipient cells (Kogure et al., 2013; Chen et al., 2016; Lang et al., 2017; Pan et al., 2017; Zhang et al., 2017). DNA recruitment and functional relevance requires further exploration (Cai et al., 2016; Kalluri and LeBleu, 2016).

Taken together, Exo/TEX characterization affirmed that Exo are important intercellular communicators allowing sessile cells a systemic crosstalk. As outlined below, there is strong evidence that CD44/CD44v6 contributes to ILV loading and via its association with tetraspanins to the recruitment of membrane integrated and membrane-attached cytosolic proteins (Wang et al., 2016).

## Linking CD44/CD44v6 activities to CIC features

CD44/CD44v6 fulfills a far wider range of activities as originally suggested for the leukocyte adhesion molecule (Idzerda et al., 1989) that is the major HA receptor (Aruffo et al., 1990). This is due to the multitude of isoforms by very variable patterns of glycosylation and the insertion of diverse combinations of alternatively spliced exons (Screaton et al., 1992). This review will restrict to provide an overview of CD44/CD44v6 activities in CIC with some reference to SC as CIC adopt many features from embryonic and adult SC. We also want to mention that in many instances a contribution of CD44 was elaborated, not taking into account the engaged CD44 isoform. This extends to reports on the engagement of CD44v6, where a contribution of this alternatively spliced exon product was approved, but expression of additional splice variant products was rarely taken into account. Despite these restrictions, there is abundant information on the contribution of CD44/CD44v6 to CIC activities, where we start with CIC inherent features.

### A niche for CIC and the contribution of CD44

During ontogeny, the fate of SC is determined by the position in a niche, composed of epithelial and mesenchymal cells and extracellular substrates that harbor growth factors, chemokines and proteases (Wolpert, 1996; Mosaad, 2014). SC niches minutely regulate SC location, quiescence/activation, symmetric/asymmetric division, and differentiation (Li and Neaves, 2006). CIC may not essentially require a niche, but survival, homing and the balance between quiescence and growth is supported by a preformed niche (Díaz-Flores et al., 2006; Li and Neaves, 2006; Hendrix et al., 2007; Morrison and Spradling, 2008). In addition, there is evidence for a contribution of CD44/CD44v6 in establishing a CIC niche.

**Figure 2:**
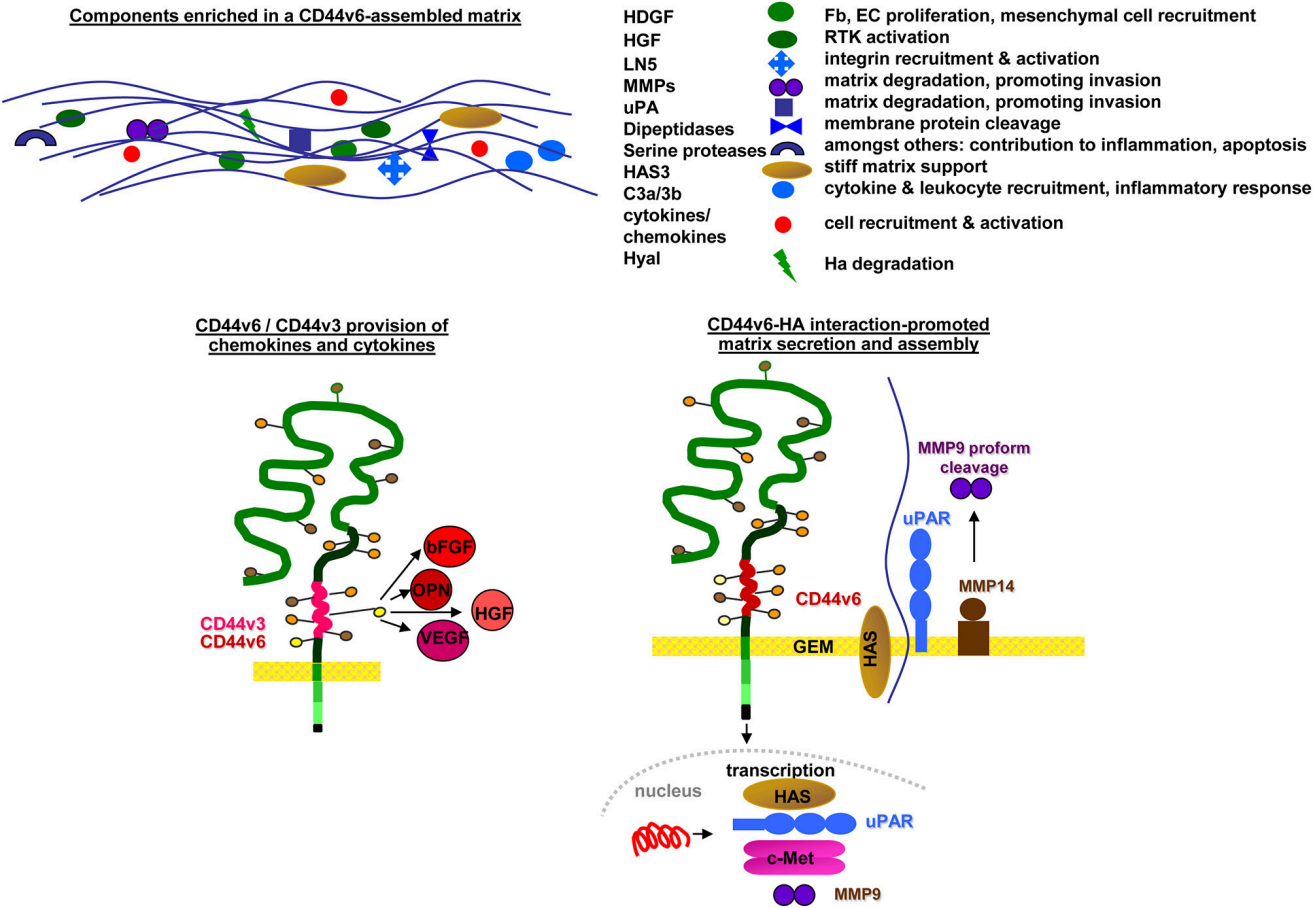
Selective contributions of CD44v in modulating a niche for SC and CIC: CD44v6 contributes to matrix assembly and modulation by transcription of HAS and several proteases. CD44v3, CD44v6, and CD44v10 are engaged in cytokine/chemokine harboring, which become deposited in the matrix. Major components of CD44v-promoted changes in matrix composition and the contribution of CD44v-promoted deposits to matrix modulation are shown.

SC niches and the tumor matrix are rich in HA and CD44 (Zhang et al., 2003) and the HA-CD44 association facilitates SC and CIC arrest (Owen and Friedenstein, 1988). However, the matrix is not only an anchor, but also affects SC/CIC by promoting changes in cell shape, intracellular tension, and gene expression (Tabe and Konopleva, 2014). The matrix-induced changes in SC/CIC, in turn, affect the composition of the matrix by promoting HA expression in niche cells (Lee et al., 2015). Furthermore, CD44v6 is engaged in matrix assembly. A CD44v6 knockdown (kd) in CIC strikingly affects the organization of the tumor matrix (Jung et al., 2009, 2011), CD44v6kd cells secreting a matrix prohibiting CIC adhesion (Oliferenko et al., 2000; Klingbeil et al., 2009). Several components contribute to the lost support for CIC adhesion. Hyaluronan synthase (HAS)3 expression is strikingly reduced in CD44v6kd cells (Jung et al., 2009), a phenomenon described in several tumors (Kultti et al., 2014). Hepatoma-derived growth factor (HDGF), too, is reduced in CD44v6kd cells (Jung et al., 2009). HDGF stimulates fibroblast, endothelial cells (EC) and vascular smooth muscle cell growth and recruits mesenchymal SC (Bao et al., 2014). Reduced clusterin secretion influences chemokine secretion and initiates stromal changes, which affect intercellular communication (Xiu et al., 2015). The complement components (C)3a and C3b, abundant in the CIC matrix, but absent in the CD44v6kd matrix, support CIC survival by inflammatory cytokine recruitment (Cramer et al., 2006). CD44v3 additionally contributes to matrix assembly. CD44v3-associated GAG recruit a large range of growth factors and chemokines, besides others HGF, fibroblast growth factor (FGF)2, OPN, and VEGF (Van Driel et al., 2002) (Figure 2). Liberation of these niche-deposited factors promotes SC/CIC arrest in the niche, but also migration and activation of signaling cascades.

Furthermore, LIC depend on adhesion to a niche in the bone marrow (BM) (Becker, 2012), which contributes to LIC maintenance (Jin et al., 2006; Funayama et al., 2010). LIC adhesion requires CD44 and HA (Christophis et al., 2011; Singh et al., 2013), such that anti-CD44, soluble HA or Hyal treatment all block settlement in the BM niche. The CD44 isoform accounting for adhesion to the BM niche was not consistently evaluated. LIC adhesion to the BM niche may rely on an embryonic CD44v isoform (Holm et al., 2015) or may profit from CD44v expression (Bendall et al., 2000; Liu and Jiang, 2006; Redondo-Muñoz et al., 2010) or differ between leukemia subtypes as elaborated for CML-like myeloproliferative neoplasia and AML LIC, which use diverse mechanisms for niche embedding (Krause et al., 2013), the majority of LIC apparently compete with HSC for the BM niche via CD44s binding. CD44/HA also is engaged in CIC homing. Patients with progressed colorectal cancer (CRC) have a lower survival rate when tumors display high HA staining (Ropponen et al., 2001). The authors suggest CD44-mediated adhesion to HA being crucial for CIC communicating with the surrounding host tissue. Similar to pancreatic (Pa)-CIC (Jung et al., 2009), urothelial and ovarian CIC also depend on CD44v6 for creating an adhesion-promoting niche (Kuncová et al., 2005; Tjhay et al., 2015); gastric CIC preferentially use CD44v8-v10 (Lau et al., 2014). The reason for the shift from preferential CD44s adhesion in LIC to CD44v in CIC are not yet elaborated. It may in part rely on epithelial cells more frequently expressing CD44v and could be supported by CD44v recruitment into GEM where clusters of CD44v-associated molecules may strengthen the interaction with the matrix (Klingbeil and Isacke, 2011). Finally, though receiving less attention, niche cells also contribute to LIC/CIC homing, where e.g., LIC poorly adhere to the BM of a CD44v7ko mouse (Christ et al., 2001).

Taken together, CIC may not essentially depend on a niche, but they profit from a niche and even compete with SC for a preformed niche as demonstrated for LIC ousting HSPC from the BM niche. CIC adhere to HA, HA binding of CIC promoting HA secretion by niche cells in a feedback loop. In addition, CIC modulate the niche composition by provision of proteases that degrade matrix proteins, including HA, by delivery of growth factors engaged in recruitment of mesenchymal SC and by activation of resident cells.

### CIC and apoptosis resistance

Poor prognosis of patients with disseminated cancer relies in part on CIC resistance toward conventional chemo- and radiation therapy (Holohan et al., 2013; Di and Zhao, 2015; Mansoori et al., 2017). CIC use several escape pathways. One mechanism builds on required resistance due to mutations in gatekeeper oncogenes such as EGFR, ALK and MET. A second pathway of drug resistance relies on DNA damage repair. DNA damage induces a cell cycle arrest that allows the cell to repair. These repair mechanisms are frequently distorted in cancer due to gain of function in oncogenes and loss of function in tumor suppressor genes. Most prominent is p53 that regulates several cell cycle checkpoints. Changes in DNA damage repair also account for resistance toward radiation, which induces double strand breaks. Cancer cells also gain in apoptosis resistance by deregulation of apoptosis signaling pathways. Cancer cells frequently express antiapoptotic proteins like Bcl2, IAP, and Flip at an increased level. These proteins are targets of the transcription factors NFκB and STAT3 that become activated during oncogenesis. Additional apoptosis resistance mechanisms rely on induction of adaptive responses and or support by the microenvironment. Finally, cancer cells make use of transmembrane proteins that account for drug efflux, most prominent ABC transporters, which eliminate hydrophobic compounds. ABC transporters are highly expressed on excretory cells like colon and pancreatic duct epithelial cells. All these mechanisms of apoptosis resistance are evoked by CIC rather than Non-CIC (Allouche et al., 2000; Bates et al., 2001). In many instances, CD44/CD44v6 contributes to or is of central importance for the apoptosis resistance of CIC, which will be discussed for CD44-promoted RTK activation of anti-apoptotic proteins (Preston and Sherman, 2011), the impact of CD44 on the metabolic state and the cooperation of CD44 with ABC transporters (Orian-Rousseau and Sleeman, 2014) and will include, where appropriate, the requirement of CD44/CD44v6 to interact with HA (Allouche et al., 2000; Fujita et al., 2002; Stern, 2008; Toole and Slomiany, 2008).

#### CD44 and receptor-mediated apoptosis

CD44–/CD44v-mediated activation of anti-apoptotic proteins is frequently initiated through the association with RTK. However, CD44v6 may also cooperate with FAS. Finally, the cooperation of CD44 with proteases contributes to apoptosis resistance.

**Figure 3:**
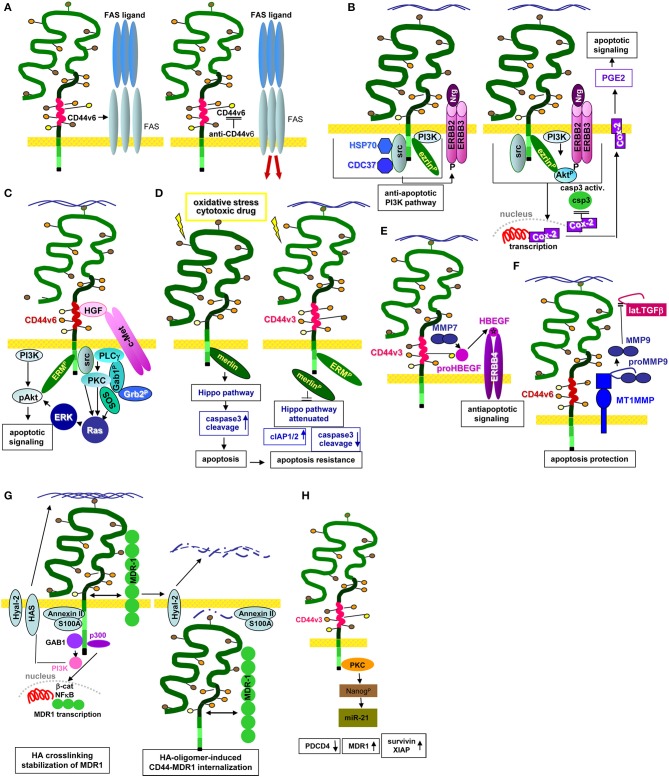
CD44/CD44v6 and CIC apoptosis resistance: **(A–H)** CD44 becomes engaged in apoptosis resistance by several mechanisms. **(A)** CD44v6 interferes with receptor-mediated apoptosis induction by preventing FAS trimerization. Antibody blocking of CD44v6 rescues FAS receptor initiated apoptosis; **(B)** Upon HA-crosslinking, CD44-associated src becomes activated, the CD44 complex which additionally contains HSP70 and CDC37 phosphorylates ERBB2/ERBB3, which binds neuregulin and activates the anti-apoptotic PI3K/Akt signaling pathways. In addition, Cox2 transcription becomes promoted. The latter stimulates PEC2 and blocks caspase 3 activation, which prohibits the execution phase of apoptosis. **(C)** Activation of c-Met via CD44v6-bound HGF promotes activation of the PI3K/Akt and the PKC-Ras-ERK pathway, activated ERK1/2 contributing to Akt phosphorylation, supporting anti-apoptotic Bcl2 and BclXl liberation. **(D)** Under stress, Merlin binds to the CD44-ICD leading to activation of the Hippo pathways that promotes caspase 3 cleavage, which initiates the execution phase of apoptosis. In the presence of CD44v3, merlin does not associated with the CD44-ICD; activation of the Hippo pathway is attenuated accompanied by increased apoptosis resistance. **(E)** CD44v3 contributes to apoptosis protection by binding proHBEGF that becomes cleaved via recruited MMP7. After loading on ERBB4, anti-apoptotic pathways become activated. **(F)** CD44v6-associated MMP14 accounts for proMMP9 cleavage. Activated MMP9 interferes with TGFβ activation, prohibiting TGFβ-promoted apoptosis induction. **(G)** Both CD44 and HA contribute to drug resistance. MDR genes are associated with CD44 and CD44 regulates expression of drug transporters by HA-activated CD44 binding to Gab1, which promotes PI3K activation. Activated PI3K stimulates HA production as well as MDR transporter expression. Alternatively, HA binding to CD44 upregulates p300 expression and its acetyltransferase activity, which sustains acetylating β-catenin and NFκB-p65. β-catenin and NFκB are cotranscription factors for MDR1. HA crosslinking stabilizes the CD44-MDR1 complex. Instead, upon Hyal activation and HA degradation the complex becomes internalized, all components including S100A and Annexin II being recovered in the cytoplasm. **(H)** Via PKC activation CD44v3 promotes Nanog phosphorylation, which induced miR-21 production accounting for MDR1 release from repression.

GEM-located CD44v6 promotes apoptosis resistance by preventing FAS trimerization upon ligand binding. An antibody blockade of CD44v6 allows for FAS trimerization and strongly increases apoptosis susceptibility (Mielgo et al., 2006), which could provide a new and interesting option in fighting CIC (Figure 3A).

One pathway of CIC apoptosis resistance relies on the association of CD44 with ERBB2 and ERBB3, which promotes heterodimerization and activation in response to neuregulin (Wang and Bourguignon, 2006; Toole and Slomiany, 2008). ERBB2 activation/phosphorylation via CD44 is strikingly HA-dependent. The lipid raft-located CD44-ERBB2/ERBB3 complex includes ezrin, the chaperones HSP90 and CDC37 and PI3K, which account for anti-apoptotic protein activation. A blockade of the HA–CD44 interaction causes complex disassembly and ERBB2 inactivation (Sherman et al., 2000). The ERBB2-PI3K/Akt-β-catenin complex additionally supports COX2 expression, which is considered as a feedback loop, COX2 suppressing caspase3 activation, strengthening HA production and promoting prostaglandin (PG)E2 expression (Ghatak et al., 2005) (Figure 3B). An ERBB2/ERBB4–CD44 complex promotes an additional feedback loop via ERK phosphorylation that stimulates HA production by HAS-1,−2, and−3 phosphorylation/activation (Misra et al., 2009). Apoptosis resistance promoted by the CD44v-MET association and initiated by CD44v3- or CD44v6-bound HGF, requires ERM binding to the CD44 cytoplasmic tail and initiates Ras-MAPK pathway activation (van der Voort et al., 1999; Bourguignon et al., 2007). Activation of the anti-apoptotic PI3K-Akt pathway and β-catenin signaling is also promoted by CD44v6-HA binding (Orian-Rousseau et al., 2007; Jung et al., 2011) (Figure 3C).

An additional important mechanism of CD44-promoted apoptosis resistance relies on the engagement of CD44 in the Hippo signaling pathway. In the absence of stress, CD44 is associated with Merlin that accounts for JNK, p53, and p21 upregulation, and YAP as well as ciAP1/2 downregulation, which promotes apoptosis via caspase3 activation. However, Merlin becomes phosphorylated and dissociates from CD44 upon CD44 activation by HA binding. After Merlin dissociation, CD44 regulates YAP expression via RhoA, which results in increased apoptosis resistance (Takahashi et al., 2010; Xu et al., 2010) (Figure 3D). In a feedback loop, activated YAP binds to the RHAMM promoter inducing RHAMM transcription (Lynch et al., 2007; Zhang Y. et al., 2014).

The CD44-RTK-promoted apoptosis resistance can additionally include proteases. CD44-recruited MMP7 cleaves the CD44v3-bound proform of HBEGF. Cleaved HBEGF binds and activates ERBB4, which signals for cell survival (Hilliard et al., 2011) (Figure 3E). An additional link between CD44v6, proteases and apoptosis resistance relies on CD44v6 ectodomain cleavage by MMP9 and ADAM10 (Kim and Jung, 2012; Hartmann et al., 2015), where CD44-associated MMP14 accounts for proMMP9 cleavage and activation (Tjwa et al., 2008). Finally, CD44-dependend apoptosis resistance can proceed via CD44-promoted MMP9 expression (Desai et al., 2009), high CD44 and MMP9 expression being associated with a poor prognosis in CLL patients (Kivisaari et al., 2010; Buggins et al., 2011). The CD44-MMP9 axis provides another means rescuing CIC from apoptosis. Activated MMP9 interferes with TGFβ activation such that TGFβ-promoted apoptosis becomes silenced (Ugarte-Berzal et al., 2014; Zarzynska, 2014) (Figure 3F).

Finally, we want to remember that owing to its GEM location, CD44v associates with non-RTK (Cooper and Qian, 2008), particularly the association with Src playing a central role linking extracellular signals to intracellular signaling pathways (Ingley, 2008) including, but not being restricted to apoptosis protection (Marhaba and Zöller, 2004). As GEM harbor CD44v rather than CD44s upregulation of anti-apoptotic genes mostly depended on CD44v expression (Bates et al., 1998; Katagiri et al., 1999; Ghatak et al., 2005; Bourguignon, 2008).

Briefly, CD44/CD44v6-RTK interaction play an important role in protecting CIC from apoptosis. This may include protease regulation, which, however, can independently support apoptosis protection. Finally, intracellular signaling frequently proceed downstream of RTK, but can also be initiated directly via CD44 associated cytosolic signaling molecules and cytoskeletal linker proteins.

#### CD44, apoptosis protection, and the metabolic state of CIC

CD44-HA binding also directly affects apoptosis resistance. This is mainly due to HA-binding-induced changes in metabolism.

SC and CIC maintain redox homeostasis by low oxygen production. This is partly mediated by a minimal metabolic rate (Suda et al., 2011; Schepers et al., 2013) and partly by generating energy via anaerobic metabolism. Maintaining a high rate of glycolysis limits reactive oxygen species (ROS) production. HIF1α, the master regulator of anaerobic glycolysis (Darzynkiewicz and Balazs, 2012), becomes stabilized under hypoxic conditions (Simsek et al., 2010) and reprograms glucose metabolism via transcriptional activation of glucose transporters, glycolytic enzymes, and metabolic regulatory enzymes, which promote the switch to the glycolytic metabolism (Kaelin and Ratcliffe, 2008). Besides HIF1α, SC dispose on additional regulatory molecules, including polycomb, DNA damage-related and anti-oxidant proteins that participate in ROS regulation (Takubo et al., 2010; Wheaton and Chandel, 2011). Finally, the CD44-intracellular domain (ICD) promotes expression of HIF2α (Nombela-Arrieta et al., 2013), aldolase c, 6-phosphofructose-2-kinase, pyruvate dehydrogenase kinase-1, and pyruvate dehydrogenase, early responsive hypoxia-related genes (Gatenby and Gillies, 2004; Bartrons and Caro, 2007; Pietras et al., 2014). This suggests CD44-ICD as a gatekeeper of aerobic glycolysis in CIC (Miletti-González et al., 2012; Nombela-Arrieta et al., 2013). The finding that neural SC, which reside undifferentiated in an HA-rich matrix, loose oxidative stress protection upon Hyal upregulation (Das and Baker, 2008), is in line with this suggestion. Also, maintenance of CR-CIC requires HIF-1α to stabilize ß-catenin and its transcriptional activity (Santoyo-Ramos et al., 2014). Notably, too, XBP1, the substrate of sensors for stress (e.g., IRE1), assembles a transcription complex with HIF1α, which promotes CD44, particularly CD44v6 expression in triple-negative breast cancers (Krishnamachary et al., 2012; Chen et al., 2014a).

Another pathway, whereby the CD44-HA axis interferes with apoptosis induction in SC relies on CD44-induced HA endocytosis, internalized HA protecting DNA from oxidants. The authors propose entrapment of iron ions, which inhibits the production of secondary oxidative species by the Fenton's reaction. Mutually not exclusive, HA directly scavenges primary and secondary ROI (Wang Z. et al., 2014). As palmitoylation and GEM recruitment of CD44 is a precondition for HA internalization (Thankamony and Knudson, 2006; Zhao et al., 2008), we suggest a preferential engagement of CD44v.

Taken together, SC circumvent stress by a low metabolic rate and energy generation via anaerobic metabolism. The main contributions of CD44 in metabolic pathway-promoted apoptosis resistance rely on its cotranscription factor activity and its engagement in HA internalization.

#### CD44 and ABC transporters

Multidrug resistance is a major obstacle in cancer therapy (Bourguignon et al., 2012a), rapid drug elimination supporting CIC survival (Kathawala et al., 2015). CD44 contributes by the crosstalk with MDR genes (Miletti-González et al., 2005; Toole and Slomiany, 2008).

CD44 associates with MDR genes and regulates their expression. The process requires HA-activated CD44 that binds Gab1, which promotes PI3K activation. Activated PI3K stimulates HA production and MDR transporter expression (Misra et al., 2005; Kathawala et al., 2015). Alternatively, HA-linked CD44 induces p300 expression and activation, which sustains MDR1 transcription by the cotranscription factors β-catenin and NFκB-p65 (Liu et al., 2009). Notably, in the presence of high MW HA, activated CD44v is predominantly recovered in GEM and is associated with ERM proteins and actin, which stabilize the CD44-MDR1 association. On the opposite, low MW HA does not promote CD44 activation and the CD44-MDR1 complex, including S100A and Annexin II, becomes internalized (Bourguignon et al., 2009b) (Figure 3G), which is accompanied by increased drug susceptibility (Slomiany et al., 2009). There remain open questions on this clinically highly relevant topic. First, the three HAS are supposed to produce HA of different size. However, the major transcription factors engaged in HAS1,−2, and−3 transcription are not defined. Second, more information on HA degradation by Hyal is required, amongst others the regulation of Hyal transcription (Negi et al., 2012; Midgley and Bowen, 2015). Taking into account that Hyal and low MW HA improve drug efficacy (Bourguignon et al., 2009b), and that HA-CD44 cross-linking regulates drug transporter expression (Misra et al., 2005; Kathawala et al., 2015), answers are urgently awaited (Sebens and Schafer, 2012; Lokeshwar et al., 2014).

Finally, apoptosis resistance can be promoted by the engagement of CD44 in EMT transcription factors, which was demonstrated for HA-bound CD44v3, where promotion of the Oct4-Sox2-Nanog complex induces miR-302 transcription (Bourguignon et al., 2012b). The CD44–HA-induced nuclear translocation of Nanog leads to miR-21 production and upregulation of apoptosis inhibitors and MDR1 (Campo et al., 2004; Bourguignon et al., 2009a) (Figure 3H).

There is ample evidence for the engagement of CD44 in MDR, which beside others is linked to MDR gene transcription. The size of HA being of central importance, further clarification of HAS and Hyal regulation are required. The CD44-promoted activation of transcription factors that regulate miRNA transcription involved in MDR and apoptosis-inhibitory genes adds an additional level of complexity. Nonetheless, several pathways of therapeutic interference are already explored like the use of ABC transporter inhibitors (Foran et al., 2016) or ABC-promoting miRNA (Safa, 2016). Lipid nanoparticles, equipped with a CD44v6-specific antibody to direct toward CIC, may shield drugs (Cavaco et al., 2017). To optimize these strategies, further studies clarifying the mechanisms, whereby HA-bound CD44 contributes to MDR gene expression, activation and stabilization are warranted.

### CD44, CIC, and EMT

Epithelial mesenchymal transition is accompanied by loss of epithelial cell polarity and cell-cell adhesion in adherens and tight junctions such that cells acquire a mesenchymal phenotype accompanied by motility. EMT is of central importance in embryogenesis, wound repair, and tumor progression, where EMT is linked to the population of CIC. EMT comprises a set of complex processes that are not yet fully unraveled. Accordingly, reports on the connection to/engagement of CD44/CD44v6 are still sporadic.

EMT is induced by signaling pathways including Notch, Wnt, and HH, which become activated by signals derived from the tumor-associated stroma, and EMT-inducing transcription factors including Snail, Slug, Zeb1, Zeb2, FOX, and Twist (Hoffman et al., 2002; Katoh, 2008; McCubrey et al., 2014). Snail, ZEB, Twist and LEF inhibit E-cadherin, claudin, occluding, and zonula occludens proteins. Upstream initiation can proceed through TGFß binding to TGFβRs. Alternatively, signaling is induced via bone morphogenetic proteins (BMP), frequently engaged in EMT induction in cancer. Trimerization of TGFßR initiates activation of SMAD signaling, but can also proceed through the PI3K-Akt or the MAPK or JNK pathways. EMT can also be induced via RTK, which initiate activation of signaling cascades that promote EMT-related transcription factor activation. Additional pathways proceed via Wnt binding to frizzled, and via JAG2 binding to NOTCH, which becomes cleaved, the NOTCH-ICD initiating NFκB, Snail, and Gata3 transcription. Finally, integrin binding to collagen I can contribute to transcription factor activation including NFκB, Snail, and LEF. There are excellent reviews covering different aspects of EMT in CIC (Gonzalez and Medici, 2014; Chang et al., 2015; Liu and Fan, 2015; Liao and Yang, 2017) and, though much is known, due to the multiple levels of interactions, many open questions remain. We will only review some EMT-related processes describing an engagement of CD44.

First to mention, Twist, Snail, ZEB, and Slug expression correlate with CD44 expression (Li and Zhou, 2011; Deep et al., 2014; Marín-Aguilera et al., 2014; Masui et al., 2014; Way et al., 2014), which was shown for Twist to proceed through β-catenin activation and the Akt pathway (Li and Zhou, 2011). Beyond linked expression, CD44 actively promotes EMT (Cho et al., 2012; Ju et al., 2014; Nevo et al., 2014; Wang D. et al., 2014; Yu D. et al., 2014; Fernando et al., 2015; Jiang et al., 2015; Shang et al., 2015; Wang et al., 2015), e.g., by inhibiting E-cadherin-β-catenin complex formation accompanied by β-catenin translocation to the nucleus (Li and Zhou, 2011). There is evidence that Notch plays a central role. It was shown that Notch-1 receptors by induction of Jagged-1 activate CD44, Slug, and Smad-3, which was prevented in the presence of DAPT, a pan-Notch inhibitor and soluble Jagged-1-Fc protein (Fehon et al., 2010). WNT signaling also promotes EMT-related transcription factor activation, upregulated SNAI1 repressing E-cadherin (cadh) (Katoh, 2017). The contribution of CD44 relies on its ability to bind to the cytoskeleton through ERM proteins. This network provides a platform for the tight association between LRP6 and Frizzled, which supports Wnt binding (Orian-Rousseau, 2015; Xu et al., 2015) and glycogen synthase kinase3β and casein kinase1γ (Orian-Rousseau and Schmitt, 2015). In addition, trafficking of the LRP6-containing vesicles from the Golgi to the membrane might be tethered through the CD44-ERM complex. This hypothesis, though still speculative, is supported by CD44 being found on coat protein complex 1 (COP1) and co-localization of CD44 with SNARE that promotes the fusion of MVB with the plasma membrane (Yu G. et al., 2014).

The contribution of CD44 to EMT induction can also proceed via miRNA regulation. In gastric cancer high level miR-106b, miR-93, and miR-25 expression is associated with CD44 expression. These miRNA repress inhibitory Smad7 promoting TGFβ/Smad signaling (Yu D. et al., 2014). MiR-34a suppresses EMT by directly targeting CD44 (Yu G. et al., 2014), whereas miR-203 suppression is essential for CR-CIC maintenance (Ju et al., 2014). Gastric-CIC overexpressing CD44 show increased expression of mesenchymal cell markers and reduced epithelial marker expression. miRNA-microarray analysis revealed significant upregulation of the miR-106b family. Smad7, a target of the miR-106b family, which inhibits TGF-β/Smad signaling was downregulated and TGF-β/Smad signal molecules were activated. Inhibition of miR-106b decreased self-renewal and invasiveness (Yu D. et al., 2014). Furthermore, HA binding to CD44v3+ALDH+ CIC in head and neck squamous cell carcinoma (HNSCC) affects the activity of the histone methyltransferase DOT1L in regulating histone modifications and miRNA activation. HA-crosslinking of CD44v3 promotes the association of Nanog/Oct4/Sox2 with CD44v3. Nanog/Oct4/Sox2 translocate and bind to the promoter sites of e.g., miR-302 and miR-21, their expression leading to stemness properties. DOT1L also becomes up-regulated and stimulates miR-10b expression accompanied by downregulation of HOXD10 and upregulation of uPAR, MMP14, and RhoC and gain in invasiveness (Bourguignon et al., 2016).

Finally, there are several reports on the engagement of the CD44-ICD in EMT induction. After cleavage of the extracellular domain. CD44 becomes accessible to presenilin, which allows for setting free the CD44-ICD that acts as a cotranscription factor (Nagano and Saya, 2004). Thus, in thyroid carcinoma cells, which harbor activated RET/PTC, RAS, or BRAF, CD44-ICD accumulated, a blockade of γ-secretase blunting CD44 processing (De Falco et al., 2012). In mammary cancer the CD44-ICD is engaged in EMT-related transcription factor expression and nuclear translocation, particularly of Oct4 and Sox2 (Cho et al., 2015) In glioma CIC, the CD44-ICD is engaged in hypoxic state maintenance via binding HIF2α, which enhances HIF target gene activation at perivascular oxygen tension (Johansson et al., 2017). Thyroid carcinoma cells harboring activated oncogenes exhibited CD44-ICD accumulation. The CD44-ICD binds to the transcription factor CREB, which increases CREB-mediated gene transcription and recruits CREB to the cyclin D1 promoter, cyclin D1 assisting CIC proliferation (De Falco et al., 2012).

On the other hand, CD44 expression also becomes regulated by EMT-related transcription factors. NOTCH1 is a critical regulator of stemness in HNSCC. The ICD promotes sphere formation and increases Oct4, Sox2, and CD44 expression. Interestingly, a NOTCH-kd decreases expression of nearly all ABC transporter genes (Lee et al., 2016). In Pa-CIC, decreasing NOTCH1 is accompanied by reduced DCLK1 (doublecortin like kinase 1), CD44, CD24, and EpC expression (Ponnurangam et al., 2016). In prostate cancer, too, NOTCH contributes to CIC maintenance. Lunatic Fringe (Lfng) encoding an O-fucosylpeptide 3-ß-N-acetylglucosaminyltransferase modifies epidermal growth factor repeats of Notch receptor proteins. Deletion of Lfng alters Notch activation and results in prostate intraepithelial neoplasia, accompanied by expansion of CD44+ and CD49f+ CIC and enhanced prostatosphere-forming capacity (Zhang S. et al., 2014). Notch activation can also be initiated via cytokines. Upon binding of the secretory cytokine prolactin (PRL) to its receptor JAK, ERK and STAT signaling becomes activated and the Notch ligand Jagged 1 expression is increased, corresponding to upregulated Notch-ICD, DCLK1, LGR5, ALDH1, and CD44 expression (Neradugomma et al., 2014). In breast cancer, Notch-ICD transactivates SOX2, which increases sphere formation, and expansion of ALDH1+ and CD44+ cells (Azzam et al., 2013). Ribosomal S6 kinase (RSK), suggested as a therapeutic target, also acts via Notch. RSK phosphorylates YB-1, which regulates CD44 and CD49f. This is due to silencing YB-1 or RSK reducing Notch4 mRNA and Notch4-ICD (Reipas et al., 2013).

Thus, CD44-ICD promotes EMT-related transcription factors and EMT-related transcription factors, particularly NOTCH contribute to CD44 expression in CIC. As elegantly reviewed, these informations offer pathways to therapeutically attack the CIC biomarker CD44/CD44v (Horta et al., 2015).

Taken together, EMT is a central feature of CIC. There is convincing evidenc for CD44v6 promoting Wnt signaling via the association with LRP5/6 and for CD44-ICD regulating EMT transcription factors. CD44, in turn, is regulated by EMT transcription factors and EMT-regulating miRNA.

## CD44/CD44v6 associations contributing to CIC motility and invasion

CD44/CD44v6 contribute to tumor cells leaving the mass of the primary tumor to circulate in blood and lymph and to settle in lymph nodes and distant organs, which is supposed to be linked to a subpopulation of CIC (Hermann et al., 2007). The engagement of CD44/CD44v6 mostly relies on its association with signal transducing molecules, predominantly receptor tyrosine kinases, GPCR and integrins. CD44/CD44v6 also affects and becomes affected by proteases, whereby the CD44-ICD comes into play. Finally, the CD44/CD44v6 interaction with the surrounding matrix, including HA, and feedback loops contributes to the regulation of CD44/CD44v6-promoted CIC motility and invasion.

### CIC, CD44v, and signaling receptor activation

CD44/CD44v6 have a strong impact on CIC motility, which relies in part on the association of CD44 with membrane-integrated kinases. In view of excellent reviews (Katoh and Katoh, 2009; Orian-Rousseau, 2015) and an abundance of clinical studies indicating a linkage between CD44v expression and CIC migration (Naujokat, 2014; Yoon et al., 2014), we only give some prominent examples for the cross-talk of CD44v with RTK, GPCR, integrins, and cytokine receptors that contribute to CIC-selective activities.

**Figure 4:**
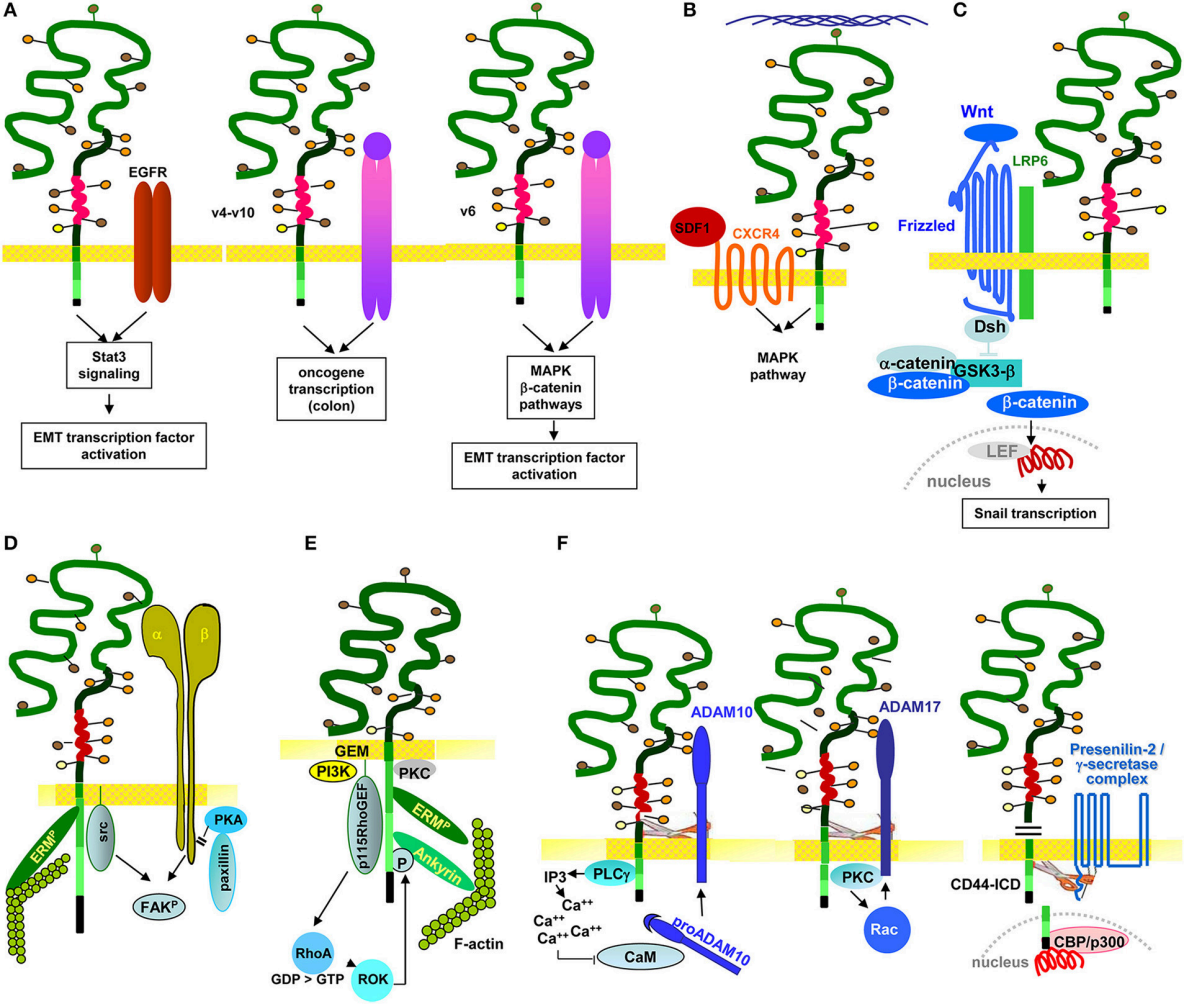
The multiple pathways of CD44/CD44v6-promoted CIC motility: CD44/CD44v6 drive CIC motility by activation of signaling cascades as well as protease activation. **(A)** Motility can become promoted by the association with RTK. CD44v-EGFR binding induces EGFR activation. The activated EGFR in cooperation with CD44-associated ERM promotes initiates activation of the Stat3 pathway and EMT gene transcription factors. Through the association of CD44v4-v10 with MET, oncogenes become activated, which is accompanied by transformation of epithelial cells in the colon. Also via the CD44v6 association with MET, ERM promotes MET and downstream MAPK and β-catenin activation, which contributes to activation and nuclear localization of several EMT transcription factors. **(B)** CD44 also associates with GPCR, a prominent example being the CD44-CXCR4 axis. Depending on HA crosslinking, SDF1-bound, CD44v-associated CXCR4 promotes activation of the MAPK pathway, which supports tumor cell and EC motility. **(C)** CD44v contributes to EMT. As demonstrated for activation of the Wnt signaling cascade, the supportive CD44 activity can rely on CD44-associated molecules. CD44-associated LRP6 binds to Frizzled, which strengthens Wnt signaling. WNT signaling upregulates SNAI1 to repress epithelial genes, such as E-cadherin. **(D)** Through the association of CD44 with integrins, CD44 gains access to focal adhesion kinase (FAK), and integrins gain access to Src kinases and ERM proteins, so that the integrin-paxillin association becomes weakened and the GEM-integrated CD44-ezrin-integrin-FAK complex moves toward the leading edge of the cell, promoting cell migration. **(E)** The connection between CD44 and cytoskeletal linker protein provides another pathway promoting motility, where RHOA plays a major role. The RHOA-specific GEF p115RHOGEF, which interacts with CD44, activates the serine/threonine Rho kinase (ROCK), a downstream target of RHOA. ROCK phosphorylates CD44, leading to enhanced ankyrin binding and guiding CD44 to the leading edge of migrating cells. **(F)** The crosstalk with proteases adds to the engagement of CD44 in motility. Three examples are shown. CD44-associated PLCγ triggers via IP3 Ca++ influx. Ca++ promotes proADAM10 dissociation from calmodulin; membrane bound ADAM10 contribution to cleavage of the CD44 extracellular regions. ADAM17 colocalizes with CD44 at Rac-regulated membrane ruffling areas and becomes activated by PKC and Rac. Finally, activated ADAM17 also promotes CD44 extracellular region cleavage. After ectodomain cleavage, CD44 becomes accessible to the presenilin/γ-secretase complex, which triggers intramembrane CD44 cleavage, setting free the CD44-ICD. The CD44-ICD binding to a DNA consensus sequence in the promoter regions of CD44 and MMP-9 gene potentiates CD44 and MMP9 transcription.

Several studies demonstrate a linkage between RTK and CD44 and LIC/CIC motility. In multiple myeloma, CD44v6 contributes to homing. Differences in homing of a stroma-dependent and a stroma-independent line revealed that only the stroma-dependent line expresses IGFR1 and CD44v6. The authors describe that BM-stroma derived IGF1 promotes IGFR1 and CD44v6 upregulation, which facilitates myeloma cell migration toward the BM niche (Asosingh et al., 2000). In SSC Axl expression correlates with CD44 and ALDH1 expression. It is associated with poor prognosis and EMT induction. Axl depletion promotes intercellular junction molecule expression and downregulates Wnt and TGFßR signaling (Cichon et al., 2014). In SCC CD44v also is associated with the EGFR and promotes EGFR phosphorylation. Interrupting this crosstalk affects tumor growth and apoptosis resistance (Perez et al., 2013), where EGFR ligation initiates via the STAT3 pathway CD44 transcription (Kim et al., 2014). Intestinal organoid cultures with inducible MET deletion revealed MET signaling regulating intestinal homeostasis, regeneration and adenoma formation. These MET activities are promoted by CD44v4-v10 (Joosten et al., 2017). In line with this finding, an elegant study on the contribution of CD44v to CIC maintenance in CRC demonstrated by a knock-in that only CD44v4-v10, but not CD44s initiates adenoma development in Apc(Min/+)mice (Zeilstra et al., 2014) (Figure 4A). There is, to our knowledge, only one report suggesting CD44 to negatively regulate RTK activities. PDGFRß interacts with TNFRI, which promotes Smad2 phosphorylation. CD44 downregulation strengthens signaling via the PDGFRß-TNFRI complex, indicating a negative regulation of the activity of this RTK complex by CD44 (Porsch et al., 2014).

In brief, particularly CD44v promotes RTK activation by provision of CD44v-bound cytokines. This initiates RTK activation and downstream signaling, which fosters oncogenesis and LIC/CIC motility directly or via EMT transcription factor activation.

The cooperation of CD44/CD44v6 mostly relies on proximity in GEM microdomains and can be supported by CD44 activation via HA binding, particularly well-described for the CD44-CXCR4 axis. To give a few, selected examples: In gastric-CIC, flow-stimulated invasion was reduced by a blockade of CXCR4, SDF1, and/or CD44 (Kingsmore et al., 2016); in HNSCC, formation of podia and cell migration essentially depend on CD44 and CXCR4 expression (Faber et al., 2013). There is evidence that particularly CD44v6 contributes to CIC migration in response to SDF1. Thus, migration along a SDF1 gradient essentially depends on CD44v6, CD44v6ko cells not migrating in response to SDF1 (Nervi et al., 2006). This also accounts for the support by C3, which is selectively trapped by CD44v6 (Takahashi et al., 2010; Jung et al., 2011). C3 drives CXCR4 into lipid rafts, the association with CD44v6 strengthening the CXCR4–SDF1 axis (Lee and Ratajczak, 2009). Finally, SDF1-CXCR4-CD44-promoted motility depends on HA size. High MW HA augments SDF1-induced CXCR4 signaling in tumor cells, which is accompanied by enhanced ERK phosphorylation and increased motility. Low MW HA or a CD44 antibody blockade efficiently inhibit these effects, indicating that HA-promoted CD44 crosslinking and CD44 activation-induced CXCR4-binding are essential for SDF1-promoted motility (Fuchs et al., 2013) (Figure 4B).

Beside CXCR4 several GPCR were described to promote CIC motility via CD44. In gastric cancer, CD44+ cells show upregulation of HH pathway proteins and HH inhibition by Smoothened (Smo) shRNA decreases spheroid and colony formation, migration, invasion, and anchorage-independent growth (Katoh and Katoh, 2009). In CRC, downregulation of the GPCR LGR5 (leucine-rich repeat containing G protein coupled receptor 5) with small interfering RNA decreases the expression of CD133 and CD44, which is accompanied by loss of spheroid growth and invasiveness (Chen et al., 2014b). GPCR sphingosine-1-phosphate receptor 3 (S1P3)-promoted tumor cell migration also involves CD44. The CD44 promoter contains ETS-1 binding sites, where S1P stimulates the binding of ETS-1 to the CD44 promoter region and induces expression and nuclear translocation of ETS-1, which requires ROCK, S1P3/ROCK up-regulating ETS-1 via JNK activity. This novel S1P3-ROCK-JNK-ETS-1-CD44 signaling cascade adds another pathway to CD44-promoted chemotactic responses (Zhang et al., 2013).

Of special importance is the linkage of CD44 to Wnt signaling. This relies on the association of CD44/CD44v6 with LRP6, which drives LRP6 toward frizzled and increases Wnt signaling (Schmitt et al., 2015). In a feedback loop, it was demonstrated that HGF, OPN and SDF-1, secreted by cells of the tumor stroma, increase CD44v6 expression in CR-CIC via Wnt/β-catenin pathway activation (Todaro et al., 2014) (Figure 4C). Notably, some cytokines also contribute to SC/CIC migration via directly binding to CD44. This was demonstrated for OPN, where CD44v6kd-HSC showed impaired migration corresponding to impaired OPN binding to CD44v6kd cells (Nilsson et al., 2005; de Barros et al., 2010). In glioma CIC, OPN-induced migration required the CD44-ICD, which enhanced CBP/p300-dependent HIF-2α activity (Pietras et al., 2014).

CD44v6 also cooperates with cytokine receptors in promoting motility. This was demonstrated for the IL6R, where anti-CD44v6 impairs CIC migration toward IL6 (Yamamura et al., 2007). Correspondingly, high expression of the IL6R on CIC is associated with poor prognosis (Kim et al., 2017).

As stated above, the proximity of CD44/CD44v6 with GPCR strongly promotes CIC motility. It is our personal point of view that HA crosslinking of CD44 besides supporting CD44 activation contributes to recruitment into GEM. These structures known as a signaling platform harboring palmitoylated and myristoylated cytosolic signaling molecules (Ma et al., 2015) will greatly contribute amplifying the signal strength upon GPCR contact with their ligands.

CIC motility is assisted by the association of CD44 with integrins. Due to activation-induced proximity, GEM-located CD44 gets access to integrin-associated focal adhesion kinase (FAK) and integrins to CD44-associated Src and ERM proteins. Thereby the integrin-paxillin association becomes weakened and the CD44-ezrin-integrin-FAK complex moves toward the leading edge (Marhaba et al., 2006). The finding was confirmed by transfection of CD49d-negative tumor cells with CD49d carrying a point mutation prohibiting phosphorylation and FAK binding and by transfection of a CD44-negative tumor line with CD44 harboring a point mutation in the ezrin binding site, or with cytoplasmic tail-truncated CD44. Ligand-binding and antibody-blocking studies confirmed that CD44-CD49d ligand-induced proximity is the prerequisite for mutual access to Src, FAK, paxillin and the MAPK pathway via lck (Singh et al., 2013) (Figure 4D). Correspondingly, a CD44kd is accompanied by Src, paxillin, FAK, c-Jun and transcription factor Sp1 downregulation, where Sp1 contributes to Src transcription. This provides an alternative pathway of CD44-promoted motility, which relies mostly on the engagement of CD44 in the JNK pathway activation (Nam et al., 2015). In line with these findings and pointing toward a special contribution of CD44v isoforms, migration of CD44v6/v7ko-HSPC toward the niche is impaired, due to the failure to bind to FN (Naor et al., 1997), FN binding promoting the association of CD44v6 with α4β1.

CD44/CD44v6 promoted CIC extravasation also is supported by the cooperation/association with adhesion molecules. CRC express CD44v isoforms are sialofucosylated on O-linked glycans alike P-selectin ligands (Hanley et al., 2006), where antibody blocking and targeted deletion confirmed the contribution of this P-selectin ligand CD44v isoform to jointly with α4β1 promoting firm adhesion followed by extravasation (Katayama et al., 2003). Breast cancer cells, too, bind to E-selection via a novel CD44v isoform containing N-linked glycans, which promotes adhesion under shear stress, the authors speculating on a particular advantage for metastasizing cell extravasation (Shirure et al., 2015).

There is no evidence for a protein-protein interaction between CD44/CD44v and integrins, the amplified migratory activity relying on proximity, which allows mutual access to downstream signaling cascades.

### CIC motility, CD44/CD44v, and the association with cytoskeletal linker proteins

CD44/CD44v6 have no signaling domains. Instead, activated CD44 associates with cytoskeletal ERM and ankyrin linker proteins, which link to the actin cytoskeleton and initiate downstream signaling cascades (reviewed in: Martin et al., 2003; Bourguignon, 2008). To provide a brief overview:

CD44-HA binding is required for the CD44 cytoplasmic tail to get access to the actin cytoskeleton via ankyrin and ERM proteins (Lokeshwar et al., 1994; Fehon et al., 2010), where Rac1 activation is central in CD44-mediated cytoskeletal reorganization. This was shown by inhibiting lamellipodia formation on HA-coated plates by CD44 antibody blocking, as well as by transfection of a dominant-negative mutant Rac1 (Oliferenko et al., 2000). Phosphorylation of the guanine exchange factors (GEF) VAV1 and VAV2 (Yu D. et al., 2014), upstream regulators of Rac1, is mediated by Src (Yu D. et al., 2014). Thus, CD44-associated Src (Cho et al., 2012) could well be a starting hub for CD44-HA crosslinking-initiated cytoskeleton reorganization. Alternatively, linking CD44 to actin could proceed through the Rho kinase (ROCK), a downstream target of RhoA, where a RhoA-specific GEF interacts with CD44. ROCK phosphorylates CD44, which enhances ankyrin binding (Bourguignon et al., 2003) and guides CD44 to the leading edge of migrating cells (Lamontagne and Grandbois, 2008) (Figure 4E). The observation that melanoma cells expressing a truncated CD44 tail cannot migrate on HA, although retaining HA-binding capacity, supports this concept (Thomas et al., 1992).

Some dominance of Rac signaling in CD44/CD44v-supported motility was also described in melanoma, where CD44v8-v10 promotes destruction of VE-cadherin junctions, which facilitates melanoma extravasation (Zhang P. et al., 2014). RacGAP1 contributed to this process as depletion of RacGAP1 or overexpression of a RacGAP1 mutant attenuates melanoma cell migration concomitantly with changes in adherens junctions. RacGAP1 promoted RhoA, FAK, paxillin activation and triggered focal adhesion formation and cytoskeletal rearrangement. The authors conclude that focal adhesion signaling downstream of RacGAP1 accounts for the breakdown of tight junctions (Zhang P. et al., 2015).

Thus, evaluating the contribution of CD44/CD44v6 in driving CIC motility at the cytoplasmic level confirmed a central engagement of Rac, Rho, ROCK proceeding to kinases engaged in focal adhesion formation.

In brief, the crosstalk between CD44/CD44v, RTK, GPCR, integrins and cytoskeletal linker proteins contributes to CIC induction. Being aware that within this review we only cursorily summarized the multiple signaling pathways whereby CD44/CD44v6 become engaged in promoting CIC motility, we want to stress one most interesting study that outlines a central role of metabolism. Fatty acid synthase (FASN), a key enzyme in lipogenesis, is significantly upregulated in many cancer. The authors demonstrate in CRC that FASN accounts for CD44, MET, Akt, FAK, and paxillin upregulation and activation. Though having no major impact on primary tumor growth, attenuation of lipogenesis completely abolished metastasis formation (Zaytseva et al., 2012).

### The contribution of the CD44/CD44v6-protease crosstalk to CIC motility

Besides associating with RTK, GPCR, integrins, cytosolic signaling molecules, and cytoskeletal linkers, CD44 is engaged in protease-promoted CIC motility.

Membrane bound MMP14 and ADAM proteases strengthen CIC motility (Duan et al., 2015). The engagement of CD44 is due to protease-promoted CD44 cleavage, where Ca++ influx triggers proADAM10 dissociation from calmodulin and ADAM10 activation. ADAM17 colocalizes with CD44 in membrane ruffles that are regulated by Rac. Rac together with PKC activate ADAM17, which cleaves CD44v (Nakamura et al., 2004; Sugahara et al., 2008). The rapid activation of membrane-integrated proteases and cleavage of CD44, which is initiated by HA-CD44 crosslinking supports motility. Notably, CD44 cleavage is tightly regulated. On the one side, activation of CD44-associated proteases by CD44 is missed after CD44 cleavage. On the other side cleavage promotes CD44 transcription. Ectodomain cleaved CD44 becomes accessible to presenilin/γ-secretase, which triggers intramembrane CD44 cleavage. The CD44-ICD binds to the CD44 and MMP-9 promoter, which potentiates CD44 (Okamoto et al., 2001) and MMP9 transcription (Miletti-González et al., 2012) (Figure 4F).

MMP9 also contributes to CD44 cleavage, inhibition of both CD44 and MMMP9 being accompanied by reduced migration. Cleavage of CD44 by MMP9 is promoted by colocalization at the cell surface and MMP9 stimulation (Chetty et al., 2012). Notably, by the impact of uPAR on integrin activity and the association of CD44 with integrins, a concomitant blockade of MMP and uPAR exerts an additive effect in prohibiting tumor cell migration (Veeravalli and Rao, 2012).

The CD44-CXCR4 association also stimulates protease-promoted migration. Stimulation of CXCR4 involves among others PKCζ (Goichberg et al., 2006), which induces MMP2 and MMP9 secretion (Peled et al., 1999). It is predominantly due to this protease-promoted distortion of the SDF1-CXCR4-CD44 axis that LIC adhesion in the niche becomes disrupted (Peled et al., 1999; Petit et al., 2002). Upregulated expression of MMP9 (Levesque et al., 2004), CD26 (Christopherson et al., 2003), cathepsin G and K, and neutrophil elastase (Petit et al., 2002) were also described fostering CIC motility (Bonig and Papayannopoulou, 2012), particularly the latter being suggested accounting for VCAM1 and FN degradation (Lévesque et al., 2001).

The contribution of intracellular plasminogen activator inhibitor (PAI)-1 deserves mentioning. TGFβ induces intracellular PAI-1 activation, high intracellular PAI-1 expression in niche-resident HSC accounting for retaining HSC in the BM niche. Inhibition of the TGFβ-PAI-1 signal increases motility and causes HSC detachment from the niche, which corresponds to high HSC motility in the absence of PAI-1. The authors point out that intracellular PAI-1 inhibits the proteolytic activity of Furin, diminishing MMP14 activity, which affected CD44, CD49d, and CXCR4 expression (Yahata et al., 2017). This interesting finding of intracellular proteolysis regulating SC and CIC migration opens a new therapeutic option.

Finally, ROCK, a major downstream target in CD44-cytoskeletal linker protein-initiated signaling, phosphorylates SLC9A1 (Na-H-exchanger1). Phosphorylated SLC9A1 activates Hyal2 and cathepsinB, which contribute to ECM degradation (Bustelo, 2000), thereby modulating the niche and creating space for migrating CIC.

The interplay between CD44/CD44v6 and proteases has not consistently been linked to CIC, although the recovery of CIC in the peripheral blood, in BM and metastasis-prone organs is well-appreciated. Nonetheless, the cleavage of CD44 by several proteases, the stimulation of protease transcription and secretion as well as the TGFβ-regulated intracellular PAI-1 activity on the one hand and the activation of Hyal and cathepsin B on the other hand suggest a major contribution of proteases to CIC motility via CD44/CD44v6 cleavage and matrix degradation.

## CIC, CD44, and TEX

TEX contribute to metastatic progression. Information so far implies CIC-TEX to transfer CIC-features into Non-CIC, to modulate the tumor stroma, to promote angiogenesis, to account for the formation of a premetastatic niche and to redirect hematopoietic progenitor maturation toward an immunosuppressive phenotype (Azmi et al., 2013; Kosaka et al., 2014; Whiteside, 2016; Sato and Weaver, 2018; Sundararajan et al., 2018; Zhang et al., 2018). Having outlined the general features of Exo biogenesis, we focus on a possible contribution of CD44 to vesicle loading and the impact of the CD44-promoted load on target structures and cells.

### The engagement of CD44v6 in vesicle loading

The majority of TEX are derived from invagination-prone membrane domains (Colombo et al., 2014). CD44v6 being located in GEM, TEX-CD44v6 can be expected to be predominantly GEM-derived, where tetraspanins are most strongly enriched in TEX (Zöller, 2009; Mathivanan et al., 2010). There was no evidence for CD44 enrichment or depletion in TEX compared to the plasma membrane. Nonetheless, TEX delivery of CD44v6kd cells is significantly decreased. This is due to CD44v6 being engaged in Tspan8 transcription by a not-yet defined transcription factor (Wang et al., 2016). Furthermore, TEX delivered by CD44v6kd cells significantly differ from CIC-TEX. This accounts for the protein as well as the miRNA profile, indicating an active contribution of internalized CD44 to the loading process.

**Figure 5:**
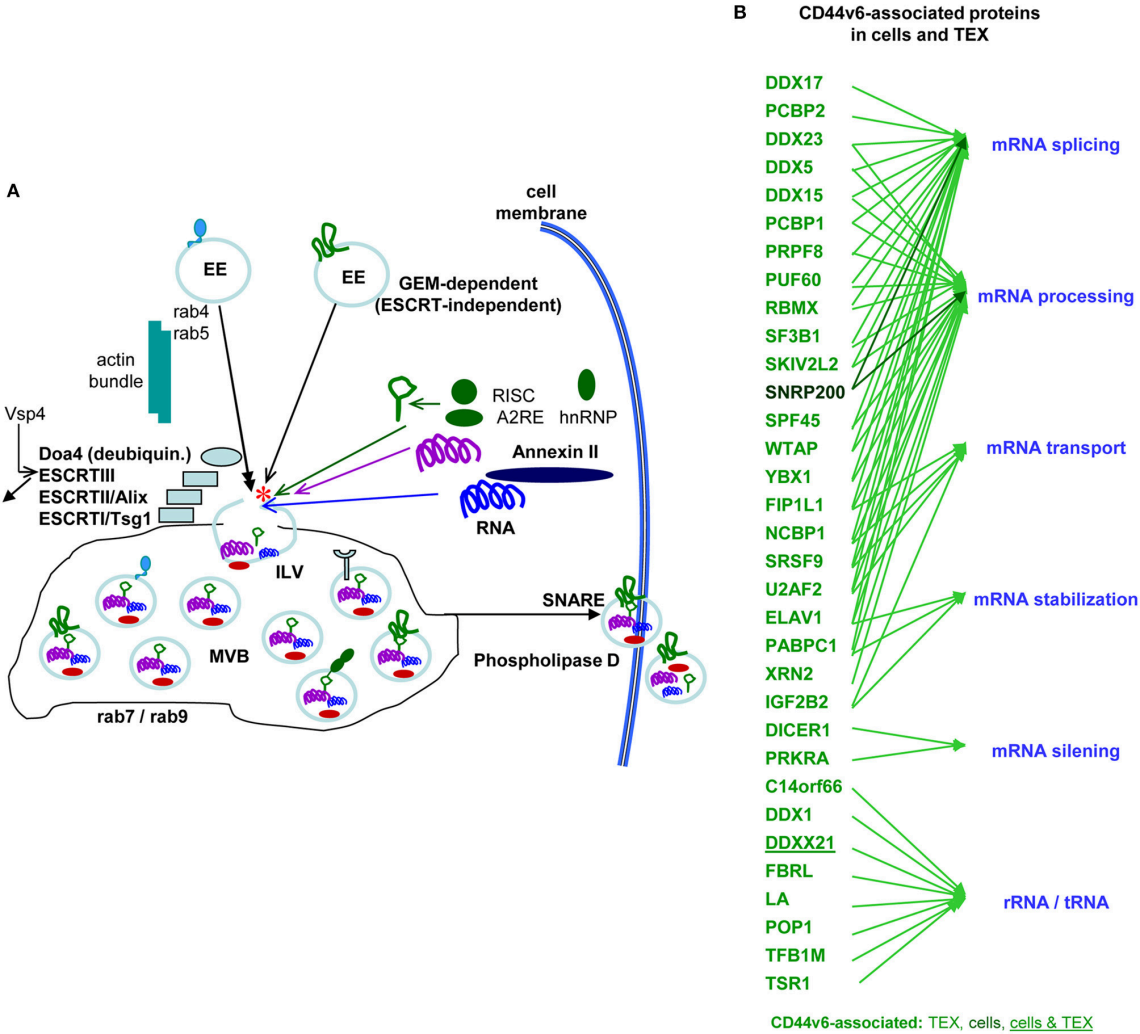
The contribution of CD44 to the biogenesis of metastasis-promoting exosomes. **(A)** Overview of Exo biogenesis, where CD44v6 contributes to loading of ILV derived from GEM-internalized membrane domains (red star). **(B)** Molecules engaged in RNA processing that coimmunoprecipitate with CD44v6, but not CD44s in CIC and CIC-TEX. With very few exceptions RNA transport and processing engaged proteins are recovered in TEX, but not cells, which supports the suggestion of an active engagement of CD44v6 in mRNA recruitment.

Earlier studies in a rat PaC model unraveled a striking decrease in MET, HAS3, HGF, HSP1, and MMP9 and a significant decrease in uPAR, CD104, PGK1 and thrombospondin (TSP) in CD44v6kd-TEX (Jung et al., 2011). While decreased recovery of MET, HGF, MMP9, uPAR and CD104 corresponds to the reduced recovery in CD44v6kd cells and likely is due to the association of these molecules with CD44v6 and/or the engagement of CD44v6 in transcription or stabilization, some non-TEM proteins, e.g., AnnII, HSP1, Monooxygenase activating protein and PGK1, are also affected, which pointed toward an engagement of CD44v6 in vesicle loading. Repeating the experiment with human CIC-TEX and CD44v6kd TEX evaluating exclusively proteins that co-immunoprecipitated with CD44s or CD44v6 confirmed these results and provided evidence for an unexpectedly high number of CD44v6-coimmunoprecipitating molecules engaged in RNA splicing, processing, transport as well as of Dicer and PRKRA (protein activator of interferon induced protein kinase EIF2AK2) engaged in mRNA silencing (deposited: Functional Proteome Analysis, DKFZ, Heidelberg, Germany, File: ZW2612) (Figure 5), strengthening the hypothesis on the CD44v6 engagement in mRNA processing. The finding is in line with Exo harboring the mRNA processing machinery and the recovery of miRNA and mRNA processing complexes in TEX (Melo et al., 2014; Geis-Asteggiante et al., 2018). Notably, loading of the vesicles with mRNA processing proteins was CD44v6-specific, not being seen, e.g., in CIC-TEX selected according to Tspan8 expression (ENA database, accession No: PRJEB25446). The mechanism accounting for the selective CD44v6-dependent recruitment remains to be explored.

In a similar study with gastric cancer cells it was noted that expression of CD97, a GPCR and member of the EGF-seven transmembrane subfamily that binds chondroitin sulfate, α5β1 and the GPI anchored CD55, becomes strikingly upregulated in Exo, which promoted lymphatic spread of tumor cells accompanied by upregulation of CD55, CD44v6, α5β1, EpCAM, and CD151. Treatment with TEX concomitantly with a soluble matrix strengthened metastasis formation (Liu D. et al., 2016).

Besides loading with proteins, the recruitment of miRNA and lncRNA has been extensively explored and is well-summarized in recent reviews (Hewson and Morris, 2016; Bortoluzzi et al., 2017; Fan et al., 2018; Salehi and Sharifi, 2018), some miRNA being preferentially recruited via CD44v6 (accession No: http://www.ncbi.nlm.nih.gov/geo/query/acc.cgi?acc=GSE34739, ENA database, accession No: PRJEB25446). Thus, non-stochastic miRNA-loading into tetraspanin-positive ILV proceeds preferentially via a ceramide-dependent pathway. Oncogenes such as Kirsten rat sarcoma viral oncogene homolog also promote selective miRNA packaging into ILV. The transfer of selectively recruited lncRNA, adds to tumor progression and EMT induction. Of particular interest for CD44 could be MALAT1 that modifies RNA alternate splicing (rev. in Hewson and Morris, 2016).

Though the recruitment of RNA processing proteins into Exo may well contribute shaping the RNA content (Melo et al., 2014; Geis-Asteggiante et al., 2018), there is a compelling hypothesis linking the invaginated membrane complex to RNA loading. The lipid composition of the Exo membrane resembles that of raft microdomains and the inward budding process involves the raft-like region of the MVB limiting membrane. The authors suggest a continuous interaction of cellular RNA with the outer (cytoplasmic) surface of MVB, the selection for incorporating RNA into ILV being based on their affinity to the raft-like region in the outer layer of the MVB membrane (Janas et al., 2015). CD44v6 being recruited into EE derived from tetraspanin-enriched membrane domains, experimental verification of this hypothesis may unravel the pathway of the selective contribution of CD44v to ILV loading with RNA.

Another pathway, whereby CD44v could become involved describes loading of ILV to be promoted by heparin sulfate proteoglycans (syndecans, SDC), syntenin and associated regulators, which include Alix, adenosine 5′-diphosphate-ribosylation factor 6, phospholipase D2 and heparanase. All these molecules support the budding of SDC-syntenin and associated cargo into the lumen of endosomes, where SDC carrying HS and sometimes chondroitin sulfate chains have numerous ligands including morphogens, adhesion molecules and growth factors, such as Wnt, FN, and FGF. By docking these factors to cognate signaling receptors, proteoglycans act as coreceptors (Friand et al., 2015). By the selective recruitment of the SDC-syntenin complex into Exo, particularly CD44v3 and CD44v6 may play a major role in ILV loading.

Great progress in proteomic and sequencing started a new era in Exo research. CD44v6 contributes by the engagement in loading ILV with mRNA processing components. Additional selective recruitments can be expected by heparin sulfate proteoglycans. Further studies being required more precisely unraveling the mode of ILV loading, the available data already suggest a major contribution of CD44v6 to CIC-TEX activities.

### The contribution of CD44v6 to tex activity

CIC-TEX can affect non-CIC, matrix and cells of the tumor stroma, EC, hematopoiesis, and the premetastatic niche (Couto et al., 2018; Zhang et al., 2018). Though TEX-CD44/CD44v6 may add to all these activities, so far this has been confirmed for matrix modulation, premetastatic niche preparation and the impact on Non-CIC and is suggested for angiogenesis, which will be briefly discussed.

A first hint that TEX-CD44v6 contributes to preparing a premetastatic niche was obtained in a rat PaC that cannot grow locally, requiring reaching the draining lymph node for growth initiation. From there it proceeds along the lymphatic route to the lung. CD44v6kd cells did not grow and rats remained tumor free. The wt tumor matrix sufficed for metastasis development (Klingbeil et al., 2009; Jung et al., 2011). Separating the matrix from the matrix-embedded TEX revealed that TEX bound preferentially LN, HA and collagen. Due to the high content of proteases, TEX efficiently degraded matrix proteins, degradation products, and the liberated growth factors and chemokines promoting tumor cell migration and proliferation (Klingbeil et al., 2009; Mu et al., 2013). Studies with human PaCIC-TEX indicated importance particularly of uPAR, MMP2, and MMP9 as well as of the CD44v6 cooperation with tetraspanins and tetraspanin-associated integrins (Wang et al., 2016). Other studies described the importance of heparanase (Thompson et al., 2013) and integrins (Hoshino et al., 2015), which may well act in cooperation with CD44v6 (reviewed in Thuma and Zöller, 2014; Sleeman, 2015; Carrasco-Ramírez et al., 2016; Heiler et al., 2016).

There is an abundance of reports on the impact of Exo on premetastatic niche formation, which originally was elaborated for LIC and the osteogenic and vascular niche in the BM (Azizidoost et al., 2014; Schepers et al., 2015), where a strong overlap between the impact of LIC and LIC-TEX on the niche was noted (Zöller, 2015), including DKK1 expression that suppresses osteogenesis and downregulation of hematopoiesis-promoting factors like SDF1, kitL and IgF1 (Kumar et al., 2018). Premetastatic niche stroma and tumor stroma are characterized mostly by changes toward fibrosis (Masamune et al., 2018). A specific contribution of CD44 remains to be explored. In concern about the cellular content of the premetastatic niche two aspects should be mentioned. First, TEX recruit cells and second, TEX reprogram resident cells. A dominantly recruited population are myeloid-derived suppressor cells (MDSC). They may become attracted via resident Mϕ stimulated by TEX-derived MIF (Costa-Silva et al., 2015). It is, however, interesting to note that TEX from non-metastatic tumors also contribute maintaining the non-metastatic state. These Non-CIC-TEX actively support recruiting cells of the innate immune system to trigger immune surveillance (Plebanek et al., 2017). The impact of TEX on niche resident cells supporting recruitment was recently described in an elegant report. The authors noted that lung epithelial cells become triggered by TEX via activation of TLR3 to secret chemokines promoting neutrophil recruitment (Liu Y. et al., 2016). In a similar study it was elaborated that TEX support TGFß, TLR and Stat3 upregulation in BMC and LNC, which is accompanied by upregulation of Notch. Furthermore, SDF1 and immunosuppressive cytokine and protease expression was increased. This accounted for Tspan8- and CD44v6-expressing TEX (Yue et al., 2015; Wang et al., 2016).

TEX-promoted angiogenesis is considered one of the central TEX activities (rev. Grange et al., 2011; Ribeiro et al., 2013), which includes an impact of transferred miRNA (Bao et al., 2018; Yukawa et al., 2018). TEX suffice for angiogenesis culminating in disseminated intravascular coagulation (Claas et al., 1998; Gesierich et al., 2006). It mostly relies on TEX-Tspan8 (Claas et al., 1998). A contribution of CD44v6 (Yue et al., 2017) is not yet conclusive as CD44v6 affects Tspan8 expression (Yue et al., 2015). TEX also suffice for EC progenitor maturation, which is accompanied by vWF, MIF, and CXCL5 upregulation promoting CCR1, VEGFR2, and VEGF expression (Nazarenko et al., 2010). Finally, many cancer preferentially metastasize via the lymphatic system and TEX activate lymphatic EC (Yue et al., 2017; Nogués et al., 2018), a contribution of TEX-derived podoplanin being suggested (Carrasco-Ramírez et al., 2016). Also TEX induce upregulation of VEGFR3 and Lyve in lymphatic vessels (Mosaad, 2014; Thuma et al., 2016). Taken together, though there is ample evidence for the contribution of TEX to angiogenesis and lymphangiogenesis, both aspects require further elaboration, particularly in concern about an explicit contribution of CD44v6, where the initiating trigger including the contribution of CD44v6-recruited miRNA remains to be explore.

Last, not least, TEX affect Non-CIC (reviewed in Atay and Godwin, 2014; Zhang X. et al., 2015; Gopal et al., 2017). First to note, as CD44 is engaged in glucose and lipid metabolism (Tamada et al., 2012; Zaytseva et al., 2012; Nagano et al., 2013) as well as the loading of TEX with miRNA (Rana et al., 2013), we want to refer to an elegant review, which outlines key elements accounting for the metabolic switch in CIC being transferred by TEX, including glucose and lipid metabolic enzymes and relevant miRNA (Alamoudi et al., 2017). The engagement of TEX-CD44/CD44v6 in EMT induction and EMT-related transcription factor induction (Wang et al., 2016) as well as the CD44v6-promoted transfer of proteins and miRNA that reprogram Non-CIC was already mentioned (Figure 5). Finally, the mode of CD44v6 activity is maintained in TEX, i.e., CIC-TEX stimulate activation of signal transduction and proteases and regulate gene transcription in Non-CIC (Wang et al., 2016). These studies, depicting single molecules/pathways require confirmation at the systemic level.

In brief, TEX are the major vehicle in the crosstalk between CIC, the host and Non-CIC. Though the direct involvement of CD44v6-TEX in angiogenesis and stroma cell modulation requires further examination, this was demonstrated for the niche, host cell recruitment and message transfer into Non-CIC. There is evidence supporting our hypothesis that the CD44v6-associated or -recruited molecules after TEX transfer provide a signaling hub. This assumption could explain, how the limited amount of proteins, coding and non-coding RNA in TEX can have such fulminate impacts on Non-CIC, the tumor stroma, endothelial cells, and the hematopoietic system.

## Conclusion and outlook

CD44 was originally described as a leukocyte marker and homing receptor. It gained great interest when CD44v6 was described contributing to tumor progression. However, only when it was noted that CD44, mostly CD44v acts as a CIC biomarker, the amazing range of activities of these molecules became apparent.

- Though by no means fully clarified, CD44/CD44v6 unequivocally contributes to niche embedding, apoptosis resistance, EMT, and tumor progression.
- CD44 covers this range of activities due to the adhesive features of its link domain, the HA binding site, the multiple glycosylation sites including proteoglycans, which promote adhesion to matrix proteins, cell membrane markers and cytokines/chemokines. The latter are particularly important in linking membrane receptors to CD44, where the cytoplasmic tail comes into play, which again displays at least three modes of action. The CD44ICD either supports directly receptor activation and/or internalization or accounts for the recruitment of signal transduction molecules via cytoskeletal linker molecules. Alternatively, the ICD is cleaved and acts as cotranscription factor. Last, not least, CD44 is associated with molecules engaged in mRNA processing. Thereby CD44v adds to miRNA generation and contributes, beside others, to tumor suppressor mRNA silencing. There are multiple feedback loops, target genes of CD44-promoted transcription / translation being engaged in CD44 regulation.
- CD44 is only involved in selected steps of Exo biogenesis. However, due to preferential location of CD44v6 in internalization prone membrane domains and the CD44v6 engagement in transcription of molecules that are central in Exo biogenesis, CD44-promoted activities are transferred into Exo. Importantly, CD44v6 contributes to loading Exo with the RNA processing machinery as well as selected RNA and miRNA.
- CD44/CD44v6-TEX cover central CIC activities. First, CD44/CD44v6-TEX play a major role in niche formation. In concert with additional TEX components, CD44/CD44v6-TEX are engaged in (lymph)angiogenesis. Importantly, CD44v6-TEX promote a shift of Non-CIC toward CIC, where the activity of CD44v in EMT transcription factor regulation and its contribution to the miRNA profile may be most relevant parameters.
- Being aware that this review largely negotiates therapeutic translation, we want to refer to some excellent reviews (Adorno-Cruz et al., 2015; Yan et al., 2015; Wang et al., 2017; Zavros, 2017) and only mention three points. First, due to the multiple activities, the efficacy of a blockade of CD44/CD44v should exceed the efficacy of a blockade of molecules with a more limited range of action like e.g., a receptor blockade; second a selective blockade of CD44v is highly recommendable as CD44v expression is far more restricted than CD44 expression, which limits the range of potential side effects, solid tumor CIC frequently expressing CD44v6; third, it is our personal view that Exo are a most suitable therapeutic, they are easy to manipulate, can be stored, distribute throughout the body and can be tailored for preferred targets, which excludes inappropriate attacks, when derived from non-transformed cells.

There remain many open questions, but there is hope that answers allow for translating into powerful adjuvant cancer therapeutics (Johnsen et al., 2014).

## Author contributions

ZW and KZ contributed to data collection (which data was presented in the review), evaluated the manuscript draft, and gave their final approval. TH provided corrections to the manuscript draft and gave his final approval. MZ planned the organization of the review, contributed to data collection, wrote the draft, and provided corrections in respect of the final version.

### Conflict of interest statement

The authors declare that the research was conducted in the absence of any commercial or financial relationships that could be construed as a potential conflict of interest.

## Abbreviations

ABC: ATP-binding cassette
AGO: argonaute
ALDH: aldehyde dehydrogenase
Alix: ALG-2-interacting protein X
ApA: alternative cleavage and polyadenylation
Arg: arginase
BclXl: Bcl2-like
bFGF: basic fibroblast growth factor
BM: bone marrow
BMP: bone morphogenetic protein
C: complement
Cadh: cadherin
CD44s: CD44 standard isoform
CD44v: CD44 variant isoforms
CIC: cancer-initiating cell
COP: coat protein complex
COX: cytochrome C oxidase
CRC: colorectal cancer
DCLK1: doublecortin like kinase 1
Dicer: ribonuclease
EC: endothelial cell
ECM: extracellular matrix
EE: early endosomes
EMT: epithelial-mesenchymal transition
ERM: ezrin-radixin-moesin family
ESCRT: endosomal sorting complex required for transport
Exo: exosomes
FAK: focal adhesion kinase
FASN: Fatty acid synthase
Fbg: fibrinogen
FGF: fibroblast growth factor
FN: fibronectin
GAG: glycosaminoglycan
GEF: guanine exchange factor
GEM: glycolipid-enriched membrane domain
GPCR: G-protein-coupled receptors
HA: hyaluronan
HAS: hyaluronan synthase
HBEGF, heparin-binding EGF-like growth factor HCV: hepatitis C virus
HGF: hepatocyte growth factor
HDGF: hepatoma-derived growth factor
HH: hedgehog
HIF: hypoxia inducible factor
HIV: human immunodeficiency virus
hnRNP: heterogeneous ribonucleoprotein
HNSCC: head and neck squamous cell carcinoma
HS: heparansulfate
HSC: hematopoietic stem cells
HSP: heat shock protein
Hyal: hyaluronidase
ICD: intracellular domain
IDO, indoleamine 2: 3-dioxygenase
IGFR1: insulin-like growth factor 1 receptor
IKK: inhibitor of NFκB
ILV: intraluminal vesicles
JAK: Janus kinase
kd: knockdown
ko: knockout
LAMP: lysosomal associated membrane protein
Lfng: Lunatic Fringe
LGR5: leucine-rich repeat containing G protein coupled receptor 5
LIC: leukemia-initiating cells
LN: laminin
lnc: long non-coding
LRP6: LDL receptor related protein6
MDR: multidrug resistance gene
MDSC: myeloid-derived suppressor cells
Mϕ: macrophage
MMP: metalloproteinase
MVB: multivesicular bodies
NFκB: nuclear factor kappa B
OPN: osteopontin
PaC: pancreatic cancer
PAI: plasminogen activator inhibitor
PDGFR: platelet-derived growth factor receptor
PG: prostaglandin
PRKRA: protein activator of interferon induced protein kinase EIF2AK2
PRL: prolactin
RISC: RNA-induced silencing complex
ROCK: Rho kinase
ROS: reactive oxygen species
RSK: Ribosomal S6 kinase
RTK: receptor tyrosine kinase
S1P3: sphingosine-1-phosphate receptor 3
SC: stem cell
SDC: Syndecan
SDF1: Stroma-derived factor 1
Ship1: inositol phosphate-5-phosphatase D
SLC9A1: Na-H-exchanger1
Smo: Smoothened
SNARE: soluble-N-ethylmaleimide-sensitive fusion protein-attachment protein receptor
SOCS: suppressor of cytokine signaling
STAT: signal transducer and activator of transcription
TEX: tumor-derived Exo
TGF: transforming growth factor
TLR: Toll like receptor
TSP: thrombospondin
VEGF: vascular endothelial growth factor
VsP: ATPase vacuolar protein sorting
ZEB: zinc finger E-box-binding homeobox.
